# Interactions between innexins UNC-7 and UNC-9 mediate electrical synapse specificity in the *Caenorhabditis elegans *locomotory nervous system

**DOI:** 10.1186/1749-8104-4-16

**Published:** 2009-05-11

**Authors:** Todd A Starich, Ji Xu, I Martha Skerrett, Bruce J Nicholson, Jocelyn E Shaw

**Affiliations:** 1 Department of Genetics, Cell Biology and Development, University of Minnesota, Minneapolis, MN 55406, USA; 2 Department of Physiology, University of Texas Health Science Center, San Antonio, TX 78229-3900, USA; 3 Department of Biology, Buffalo State College, Buffalo, NY, USA; 4 Department Biochemistry, University of Texas Health Science Ceneter, San Antonio, TX 78229-3900, USA

## Abstract

**Background:**

Approximately 10% of *Caenorhabditis elegans *nervous system synapses are electrical, that is, gap junctions composed of innexins. The locomotory nervous system consists of several pairs of interneurons and three major classes of motor neurons, all with stereotypical patterns of connectivity that include gap junctions. Mutations in the two innexin genes *unc-7 *and *unc-9 *result in identical uncoordinated movement phenotypes, and their respective gene products were investigated for their contribution to electrical synapse connectivity.

**Results:**

*unc-7 *encodes three innexin isoforms. Two of these, UNC-7S and UNC-7SR, are functionally equivalent and play an essential role in coordinated locomotion. UNC-7S and UNC-7SR are widely expressed and co-localize extensively with green fluorescent protein-tagged innexin UNC-9 in the ventral and dorsal nerve cords. A subset of UNC-7S/SR expression visualizes gap junctions formed between the AVB forward command interneurons and their B class motor neuron partners. Experiments indicate that expression of UNC-7S/SR in AVB and expression of UNC-9 in B motor neurons is necessary for these gap junctions to form. In *Xenopus *oocyte pairs, both UNC-7S and UNC-9 form homomeric gap junctions, and together they form heterotypic channels. *Xenopus *oocyte studies and co-localization studies in *C. elegans *suggest that UNC-7S and UNC-9 do not heteromerize in the same hemichannel, leading to the model that hemichannels in AVB:B motor neuron gap junctions are homomeric and heterotypic.

**Conclusion:**

UNC-7S and UNC-9 are widely expressed and contribute to a large number of the gap junctions identified in the locomotory nervous system. Proper AVB:B gap junction formation requires UNC-7S expression in AVB interneurons and UNC-9 expression in B motor neurons. More broadly, this illustrates that innexin identity is critical for electrical synapse specificity, but differential (compartmentalized) innexin expression cannot account for all of the specificity seen in *C. elegans*, and other factors must influence the determination of synaptic partners.

## Background

Gap junctions mediate intercellular communication through diffusion of small (1 kDa) molecules. Single gap junction channels are formed from half channels (hemichannels) contributed by apposing cells. The six subunits forming a hemichannel may be homomeric or heteromeric, and the hemichannels in one channel may be identical (homotypic) or not (heterotypic). Until recently it was thought that all vertebrate gap junction subunits are connexins, and invertebrate gap junction proteins are innexins [1,2]. Although connexins and innexins share functional properties, they are likely unrelated evolutionarily [1,3]. Members of a third protein family, the pannexins, were recently shown to form gap junction channels [4]. Pannexins are found in vertebrates and lower chordates [5,6] and share sufficient sequence similarity with innexins to be considered related. Individual connexin, innexin and pannexin proteins have all been shown to form gap junction channels with similar properties as assayed in paired *Xenopus *oocytes (for example, [4,7,8]).

Previously we showed that the innexin *unc-7 *gene is essential for coordinated locomotion in *C. elegans *[9]. We are using *unc-7 *as a means to understand how electrical synapses are specifically established between cell partners. Electrical synapses are widespread in invertebrates and vertebrates [10-12]. Approximately 600 electrical synapses and 5,000 chemical synapses were described in the reconstruction of the *C. elegans *non-pharyngeal nervous system [13]. Although most neurons make electrical synapses, they do not form gap junctions with all available neighbors; little is known about how such selectivity is achieved. Because subunit composition can influence the docking between hemichannels [14], it might be possible to pattern synaptic connections through selective expression of innexins, of which 25 exist in *C. elegans *(reviewed in [1]). Alternatively, electrical synaptic specificity might be determined through other means, such as activity dependence.

**Figure 1:**
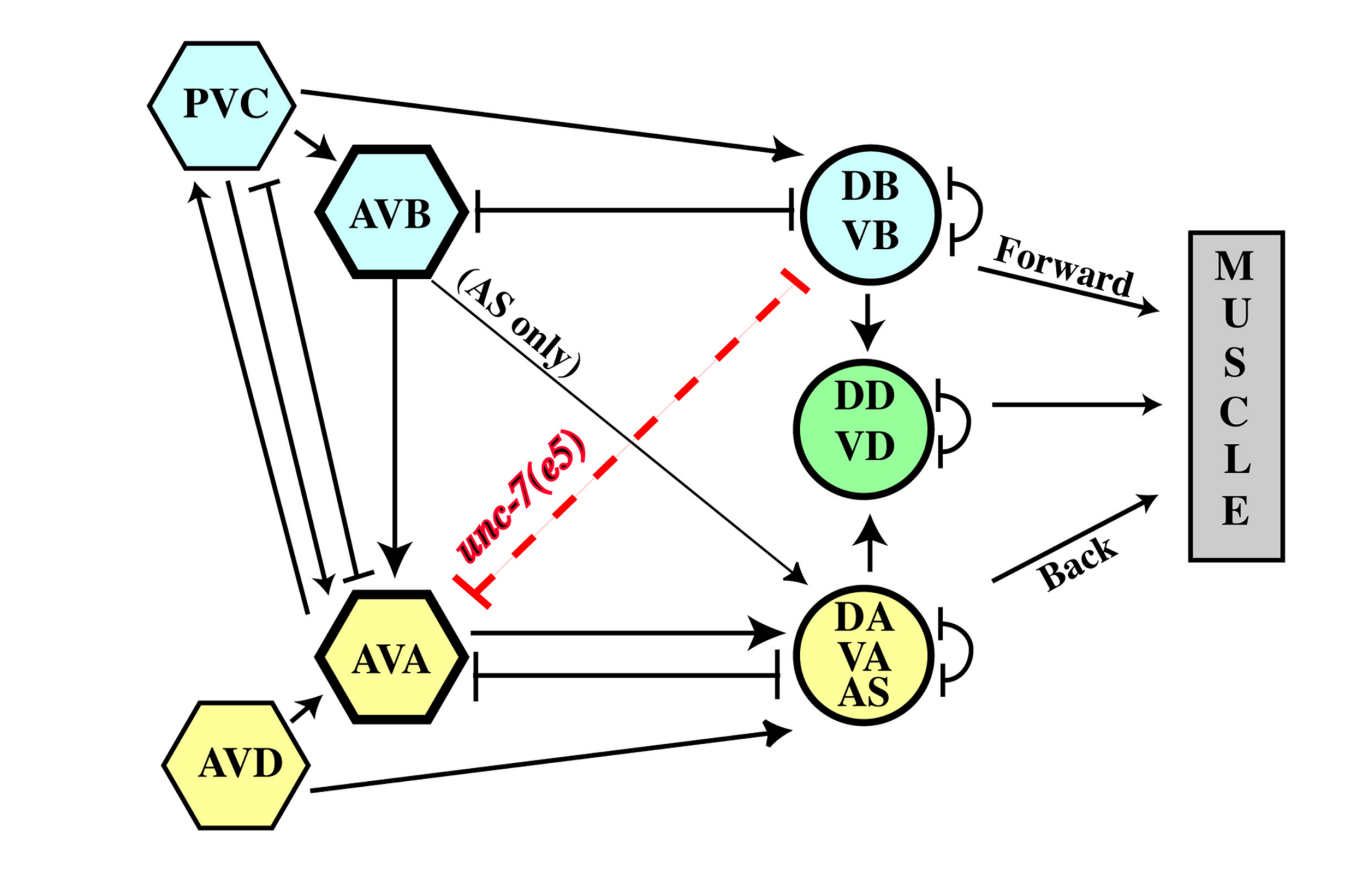
***unc-7(e5) *wiring defect and *unc-7 *point mutations**. Wiring diagram for coordinated locomotion (adapted from [13,16]) shows chemical (arrows) or electrical (terminated lines) synapses among interneurons (hexagons) and motor neurons (circles) implicated in forward movement (blue), reverse movement (yellow), or movement in both directions (green). Ectopic *unc-7(e5) *AVA:B motor neuron gap junctions are indicated (red dashed line).

A detailed model for coordinated locomotion provides a framework that makes *C. elegans *well suited for examining issues of synaptic target recognition and function (reviewed in [15]). Changes in locomotion reflect the final output for tactic, avoidance, and mating behavior. Accumulated observations from serial electron micrographs (EMs) [13], laser ablations of specific neurons [16,17], localization of excitatory and inhibitory neurotransmitters [18,19], and electrophysiological studies in the similar nervous system of *Ascaris suum *[20] have contributed to this model (Figure 1). Ablation of the AVB interneuron pair results in a forward Unc (uncoordinated) phenotype. AVBs form gap junctions with target dorsal and ventral nerve cord excitatory B class (DB and VB) motor neurons. Ablation of AVA interneurons results in a backward Unc phenotype, and AVAs form both gap junctions and chemical synapses with excitatory A class (DA, VA, AS) motor neuron targets. D class motor neurons receive input from excitatory motor neurons in either the dorsal or ventral nerve cord and inhibit contralateral muscles. PVC and AVD interneurons are considered modulatory – ablation of either interneuron pair alone has little effect on locomotion, but phenotypes are exacerbated if co-ablated with corresponding command interneurons (PVC with AVB, AVD with AVA) [16]. Motor neurons may form intra-class gap junctions or gap junctions to others in the same 'compartment' (forward, reverse, inhibitory), but they rarely form gap junctions with motor neurons of another compartment, though their processes lie in close proximity [13].

*unc-7 *mutants are severely uncoordinated. Instead of propagating smooth sinusoidal waves they kink. Forward and backward movement are both affected, but the forward Unc phenotype is more severe. The cause of the Unc phenotype is unknown but could be due to loss of functional gap junctions or to extra ectopic gap junctions established between AVA interneurons and B motor neurons, noted in EM serial sections of an *unc-7(e5) *animal (White *et al*., personal communication, originally cited in [9]; Figure 1). The related *unc-9 *innexin gene [21] has an Unc phenotype similar to that of *unc-7*, but no EM reconstruction data for *unc-9 *are available.

To gain an understanding of how electrical synapses specifically form between neurons, we are interested in determining which gap junctions identified in the reconstruction of the nervous system have UNC-7 as a component, which other innexins contribute to these junctions, what determines specificity for forming complete channels, and how these gap junctions function to direct information flow in the nervous system. We demonstrate that the formation of specific gap junctions between AVB and B motor neurons depends on interactions between *unc-7 *and *unc-9 *gene products, and that the expression of *unc-7 *and *unc-9 *influences formation of a large proportion of the gap junctions in the locomotory nervous system.

## Results

### *unc-7(e5) *mutants lack functional gap junction channels

Since *unc-7 *encodes a gap junction protein, we presumed that the mutant Unc-7 uncoordinated phenotype reflects a loss of functional gap junction channels. However, partial EM reconstruction of an *unc-7(e5) *mutant revealed ectopic gap junctions formed between AVA interneurons that direct backward movement and B class motor neurons involved in forward movement. No other connectivity changes were noted. Therefore, part of the characterization of the Unc-7 phenotype involves understanding how ectopic gap junction channels arise in the absence of the UNC-7 innexin, and whether or not these ectopic neuronal connections contribute to the Unc phenotype.

**Figure 2:**
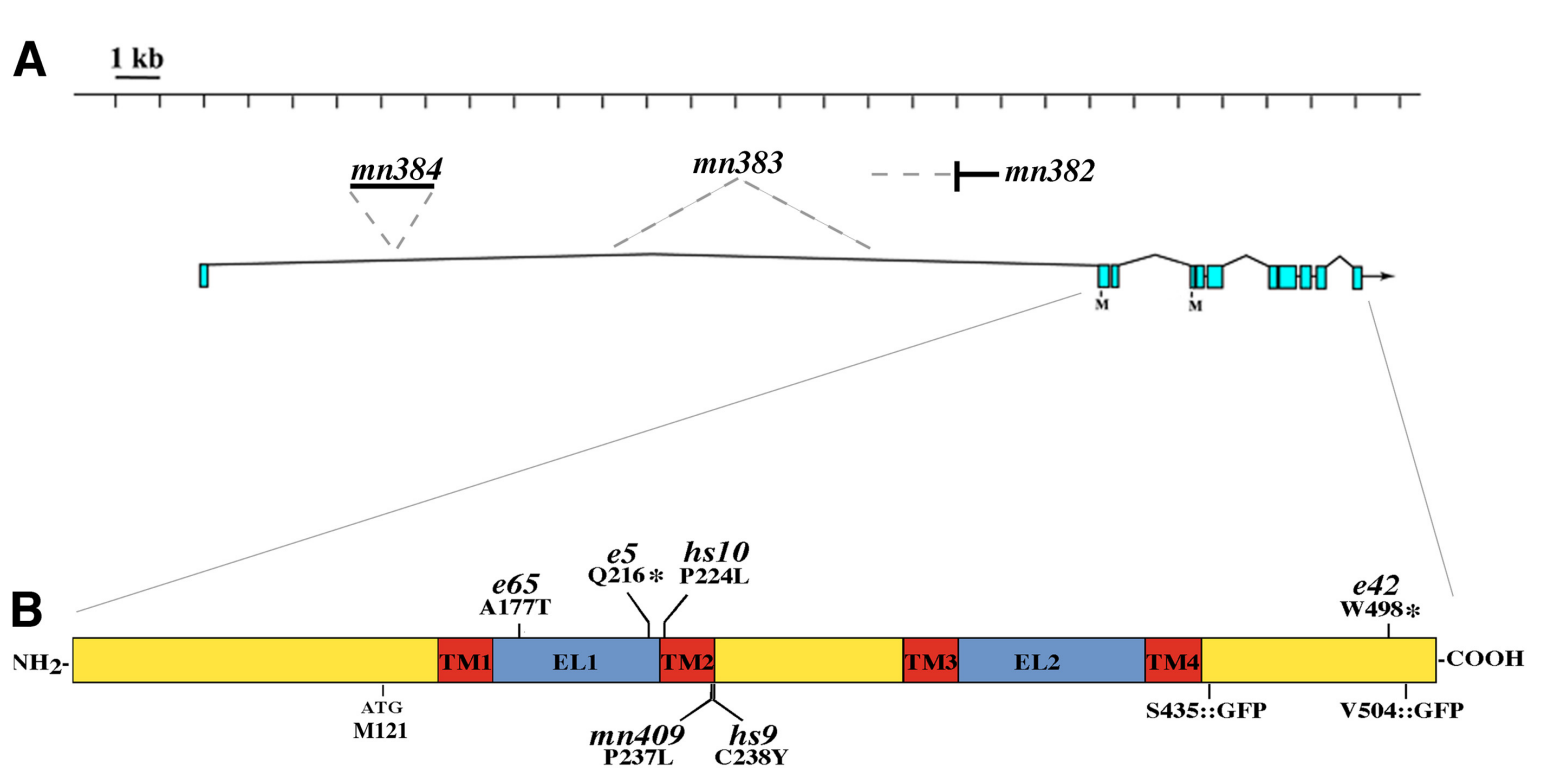
***unc-7 *mutations**. **(A) **The *unc-7 *locus showing the approximate sites of rearrangements within intron 1 resulting in an Unc-7 phenotype. *mn382 *is a translocation, *mn383 *is a 6-kb deletion, and *mn384 *is a 2-kb insertion. **(B) ***unc-7 *point mutations and their location in the largest UNC-7 isoform (UNC-7L, 522 amino acids). ATG at M121 is the conserved innexin initiation site and start site of UNC-7SR. The position of GFP in *unc-7S::gfp *is also indicated. Asterisks indicate stop codons. EL, extracellular loop; TM, transmembrane domain. (The cold sensitive *unc-7(hs10*) allele was originally used as the basis for defining the gene *unc-124 *[50], and a heteroallelic interaction with *unc-7 *was reported [21,50]. Since *hs10 *is a mutant allele of *unc-7*, the existence of *unc-124 *is dubious. See Materials and methods for details.)

Ectopic gap junctions might form if wild-type UNC-7 acts to restrict neuronal gap junction formation in some manner, or they could arise due to neomorphic or hypermorphic activity of a mutant UNC-7 protein that initiates formation of new gap junction connections. DNA sequencing of *unc-7 *mutants revealed that the molecular lesion associated with the *unc-7(e5) *allele is a premature stop (Q216*) in the first predicted extracellular loop (EL1) of UNC-7 (Figure 2B; Table 1). *e5 *therefore represents a probable null mutation, and ectopic gap junctions seen in *unc-7(e5) *animals are not the result of UNC-7 over-expression. A truncated *e5 *product might interfere with channel formation and mimic a loss of function mutation, but it seems unlikely that a product lacking extracellular domains could initiate formation of ectopic gap junctions. Therefore, we conclude that the appearance of ectopic gap junctions in *unc-7 *mutants is due to loss of the UNC-7 product, consistent with the recessive nature of the *e5 *allele.

**Table 1:** *unc-7 *alleles

Allele	Mutagen	Molecular lesion	Phenotype	Anti-UNC-7 staining pattern
*e5*	EMS	Q216*(CAA→TAA)	Severe	No staining
*e42*	EMS	W498*(TGG→TAG)	Severe	No staining
*e65*	EMS	A177T (GCC→ACC)	Weak	Wild-type staining
*e133*	EMS	Undetected	Moderate	Strong nr, rvg; weak vnc, dnc
*e139*	EMS	Undetected	Moderate	Strong nr, rvg; weak vnc, dnc
*hs9*	EMS	P224L (CCG→CTG)	CS	Weak signal, mostly cytoplasmic
*hs10*	EMS	C238Y (TGC→TAC)	CS	Wild-type staining
*mn382*	γ-Ray	Translocation; break point in intron 1	Severe	Strong nr, rvg; weak vnc, dnc
*mn383*	γ-Ray	6-kb deletion, intron 1	Severe	Strong nr, rvg; weak vnc, dnc
*mn384*	γ-Ray	2-kb insertion, intron 1	Weak	Wild-type staining
*mn409*	EMS	P237L (CCA→CTA)	Severe	Weak signal, mostly cytoplasmic

CS, cold-sensitive; dnc, dorsal nerve cord; nr, nerve ring; rvg, retrovesicular ganglion; vnc, ventral nerve cord. EMS, ethyl methanesulfonate.

One hypothesis for *unc-7 *uncoordination is that ectopic AVA:B motor neuron gap junctions interfere with forward locomotion. If ectopic gap junctions are the sole cause of the Unc-7 phenotype, then laser ablation of AVAs should eliminate communication between these interneurons and the B motor neurons and restore normal forward locomotion; in conjunction with AVA ablation, animals should be backward Unc (Figure 1). We targeted AVA interneurons for ablation in ten wild-type L1 stage animals. Eight animals displayed a severe backward Unc phenotype but were still capable of forward locomotion, confirming that AVAs had been properly ablated. The AVA interneurons in ten *unc-7(e5*) L1 animals were then targeted; none displayed improvement in forward locomotion, supporting the conclusion that ectopic AVA:B gap junctions are not the sole cause of the Unc-7 phenotype. Therefore, the Unc-7 locomotory defect reflects loss of *unc-7 *gap junction channel function, regardless of ectopic gap junction formation, and we sought to determine which gap junctions in the nervous system include UNC-7 as a component.

### Two isoforms of UNC-7 rescue locomotion

**Figure 3:**
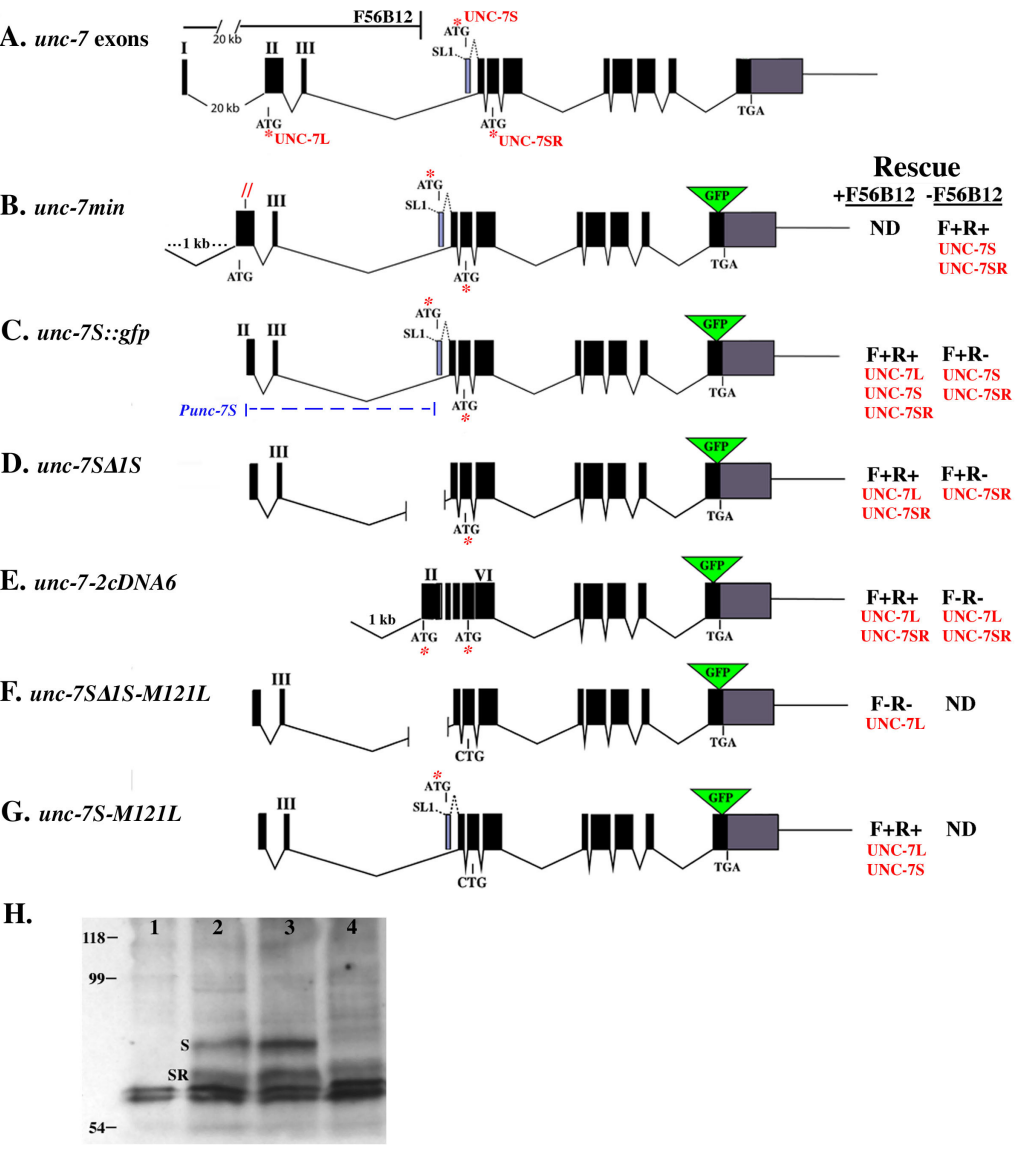
***unc-7 *transformation rescue**. **(A) **Exon/intron structure of *unc-7*. Cosmid F56B12 includes presumptive upstream promoter sequences and ends within intron 3. ATG indicates UNC-7L (exon 2), UNC-7S (exon 1S, blue shading), and UNC-7SR (exon 5) start sites. Recombination *in vivo *between F56B12 and plasmid constructs reconstituted the *unc-7 *locus. Rescue of forward (F) or reverse (R) locomotion by plasmid constructs, with or without co-injection of F56B12, was determined (isoforms predicted to be expressed are listed in red). Rescue was determined from at least three lines, except (G) (single line generated), and (F) (no rescued lines obtained). **(B) **Fully rescuing *unc-7 min *plasmid expressing UNC-7S and UNC-7SR. Red slashes indicate frameshift mutation eliminating UNC-7L expression. **(C) ***unc-7S::gfp *construct lacks UNC-7L translational start site and shares 1.4-kb with F56B12. **(D) ***unc-7SΔ1S *lacks exon 1S. **(E) ***unc-7-2cDNA6 *with exons 2–6 replaced by corresponding cDNA sequence, shares 1 kb of intron 1 overlap with F56B12. **(F) ***unc-7SΔ1S-M121L *cannot initiate translation of UNC-7S or UNC-7SR. **(G) ***unc-7S-M121L *intiates translation of UNC-7L and UNC-7S with the M121L mutation. **(H) **Western blot using anti-GFP antibodies detecting UNC-7::GFP isoforms. Predicted sizes of UNC-7L::GFP, -S::GFP, and -SR::GFP are 85, 78, and 72 kDa, respectively. Lane 1, wild type (N2); lanes 2 and 3, *unc-7(e5) *rescued with *unc-7S::gfp *+ cosmid F56B12, expected to express all isoforms; lane 4, *unc-7(e5) *rescued with *unc-7SΔ1S *+ F56B12, predicted to express UNC-7L:: and SR::GFP. ND = not determined.

UNC-7 [Swiss-Prot:Q03412] is unusual in the innexin family in having a predicted amino terminus extending 120 amino acids upstream of the canonical innexin translational start site (M121; Figure 2B). These 120 amino acids are completely conserved (two conservative substitutions) in *Caenorhabditis briggsae *[22]. *unc-7 *includes a 20-kb intron following the first (non-coding) exon; mutations confined to intron 1 (Figure 2A) confer a strong (*mn382 *and *mn383*) or weak (*mn384*) Unc-7 phenotype [9]. Northern analysis of these mutants indicated that more than a single transcript might be produced from the *unc-7 *locus [9]. 5' Rapid Amplification of cDNA ends (RACE) analysis identified a second *unc-7 *transcript with an alternative exon 1 (exon 1S; Figure 3) encoding a novel translational start site. The predicted product (UNC-7S) includes 52 amino acids in the amino terminus upstream of M121, 14 of which are unique to UNC-7S; this sequence is completely conserved in *C. briggsae*. Various constructs were designed to examine possible roles for UNC-7S and the predicted full-length *unc-7 *product (UNC-7L). The *unc-7 *genomic region exceeds 25 kb; in order to generate a full-length *unc-7 *locus, some transformation rescue experiments relied on recombination between the cosmid F56B12 (encoding the presumptive promoter region of *unc-7 *extending into the third intron; Figure 3A) and plasmids containing the *unc-7 *coding region. An inserted green fluorescent protein (GFP) tag replaced the carboxyl-terminal 17 amino acids of UNC-7; addition of GFP did not appear to affect function. Our results, summarized as follows, indicate that UNC-7S and a smaller isoform (UNC-7SR) are functionally equivalent and rescue all *unc-7 *locomotion defects; a role for UNC-7L has not been discerned.

The shortest genomic region capable of rescuing forward and backward locomotion defects in *unc-7 *mutants encompassed the coding region of *unc-7 *and 1 kb of promoter sequence upstream of exon 2 (*unc-7 min*; Figure 3B). This construct included a frame-shift mutation within the unique amino terminus of UNC-7L that eliminated its production, indicating that UNC-7S alone is sufficient for *unc-7 *function. A shorter version of *unc-7 min *lacked promoter sequences upstream of exon 2 (*unc-7S::gfp*; Figure 3C) and rescued forward but not backward locomotion, suggesting that promoter sequences in intron 3 (*Punc-7S*; Figure 3C) drive a subset of UNC-7S expression sufficient to rescue only forward movement. When co-injected with F56B12, the *unc-7S::gfp *construct rescued both forward and reverse locomotion.

Evidence for a second functional isoform of UNC-7 was obtained. UNC-7S expression was eliminated by deleting the UNC-7S translational start site in exon 1S (*unc-7SΔ1S*; Figure 3D). Like *unc-7S::gfp*, this construct rescued forward locomotion in the absence of cosmid F56B12, and we postulated that M121 in exon IV (the canonical innexin start site) might be used to generate a shorter but functional UNC-7S-like product (UNC-7SR).

Western blot analyses were consistent with this interpretation. Using anti-GFP antibodies, two major protein bands, not present in wild-type, were detected in *unc-7(e5) *animals rescued with *unc-7S::GFP *+ F56B12 (Figure 3H). These bands approximated the sizes predicted for UNC-7S::GFP and UNC-7SR::GFP. In animals rescued with *unc-7SΔ1S *+ F56B12, predicted to express UNC-7SR::GFP but not UNC-7S::GFP, only the smaller band was detected.

Inclusion of F56B12 in rescue experiments allowed for translation of UNC-7L::GFP, but no corresponding protein band was identified on western blots. To determine if UNC-7L might have functional activity, we tried to eliminate expression of predicted UNC-7S and UNC-7SR isoforms. The *Punc-7S *promoter sequences in intron 3 were eliminated by substituting this region with *unc-7 *cDNA sequence from exons 2–6 (*unc-7-2cDNA6*; Figure 3E). This construct (plus F56B12) fully rescued *unc-7(e5)*, suggesting that either UNC-7L can rescue locomotion, or UNC-7SR is produced through internal translation initiating at M121. M121 was mutated to L in *unc-7SΔ1S*; this construct (*unc-7SΔ1S-M121L*; Figure 3F) plus F56B12 failed to rescue *unc-7*, suggesting that UNC-7L does not have rescuing activity. To demonstrate that mutation of M121 did not lead to loss of an essential protein function, exon 1S was added back to allow for expression of UNC-7S(M121L), and this construct (*unc-7S-M121L*; Figure 3G) fully rescued. Therefore, *unc-7 *locomotion defects can be rescued by either UNC-7S or UNC-7SR, but UNC-7L cannot substitute for this function (although it may have a subtle role or function in another process). Furthermore, it appears that UNC-7SR can be produced by internal translation (Figure 3E). We will refer to the predicted products of the *unc-7 *rescuing transcript as UNC-7S with the understanding that UNC-7SR is also translated.

### UNC-7 is expressed throughout the nervous system

**Figure 4:**
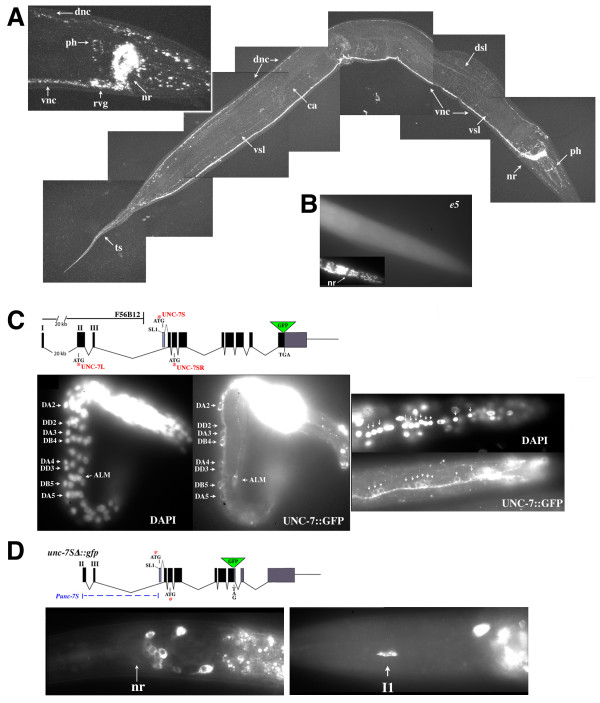
**UNC-7 expression**. **(A) **Wild-type adult stained with affinity-purified anti-UNC-7. Composite of confocal images. **(B) **Anti-UNC-7 staining of *unc-7(e5) *mutant; inset shows DAPI stain of nuclei. Anterior to right. **(C) **Early L1 larva with rescuing *unc-7::gfp *(plus F56B12) in *unc-7(e5*), anterior upper right. Right: posterior ventral nerve cord in rescued L3 animal (anterior left). Neurons expressing *unc-7::gfp *include members of all motor neuron classes (AS9-11, DD5, VD10-11, VA10-11, VB11, DB7, and DA7). **(D) **Expression of non-rescuing *unc-7SΔ::gfp *(no cosmid). I1, pharyngeal neuron I1. Anterior to left. Abbreviations: ca, canal-associated process; dnc, dorsal nerve cord; dsl, dorsal sublateral process; nr, nerve ring; ph, pharyngeal nervous system; rvg, retro-vesicular ganglion; ts, tail spike; vnc, ventral nerve cord; vsl, ventral sublateral process.

We used specific antibodies and GFP fusions to examine UNC-7 expression. Affinity-purified antibodies specific to the unique UNC-7 carboxyl terminus were generated. In wild-type animals, UNC-7 (representing all isoforms) is expressed throughout the nervous system (Figure 4A), as well as in body muscles, particularly in the posterior. Previous genetic mosaic analysis showed that the focus of action of *unc-7(+) *for locomotion is in the ABp lineage, which gives rise to nearly all of the ventral nerve cord motor neurons and the AVB interneurons. Absence of UNC-7 from muscle did not lead to an Unc-7 phenotype [9]. Total UNC-7 is often localized to puncta that may represent gap junctions. Expression of UNC-7 in various *unc-7 *mutants was examined (Table 1; Additional file 1); severe mutants *e5 *and *e42 *lack the antigenic site and showed no anti-UNC-7 reactivity, confirming antibody specificity (Figure 4B).

The anti-UNC-7 antibodies showed strong reactivity to puncta distributed throughout the nervous system that might correspond to gap junctions; however, cell bodies were rarely marked. Transformation rescue of *unc-7 *mutants, effected by the introduction of DNA constructs in multiple copies on extrachromosomal arrays, was used to assay the expression patterns of UNC-7 transgenes. In animals rescued with arrays representing reconstitution of the entire *unc-7 *locus (*unc-7S::gfp *+ F56B12; Figure 3C), UNC-7::GFP expression mirrored that observed with anti-UNC-7 antibodies. However, some cell bodies could now be recognized with variability, probably due to overexpression from the rescuing array. The strongest signals appeared late in embryogenesis and in L1 animals, coinciding with *unc-7 *mRNA levels [9], and UNC-7::GFP was detected in all motor neuron classes in the vental nerve cord (Figure 4C and Table 2); in older larvae expression was seen in all post-embryonically derived motor neuron classes (VA, VB, AS, VD, and VC; VC motor neurons are implicated in egg-laying [13,19]). Because in these animals UNC-7L::GFP expression was not detected by western blot analysis (Figure 3H, lane 2), we attribute the majority of this signal to UNC-7S::GFP and conclude that UNC-7S is broadly expressed in many interneurons and motor neurons

**Table 2:** Neurons identified by total UNC-7 or UNC-7::GFP expression

Neuron	Gap junction partners*
AVA	ASn, DAn, VAn, SAB, PVC, RIM, URY, AVA, (DB5)
ASn^†^	AVA, VAn
DAn^†^	AVA, VAn, (SAB, PHC)
DBn^†^	AVB, DBn, VBn
DDn^†^	DDn, VDn
VAn^†^	AVA, ASn, VAn, (PHC, VB11)
VBn^†^	AVB, DBn, VBn
VCn^†^	VCn, (vm2)
VDn^†^	VDn, DDn, PVP (VD1 only: SMDDR, VB1, VB2, RIFL, RIGL, DVC)
ADL^†^	OLQ, RMG
ALM^†^	AVM, PVR, (AVDR)
AVM^†^	ALM, AVD
AVG	RIF
DVC^†^	AVL, PVP, VD1
PDE	PVC, PVM, VD9
PVD^†^	None described

*From White *et al*. [13]. ^†^Not detected with UNC-7S constructs. Gap junction partners in parentheses may form an occasional gap junction with the associated neuron.

Expression of UNC-7S driven by the *Punc-7S *promoter was previously found to be sufficient to rescue forward but not backward locomotion (Figure 3C). To try to identify the subset of *unc-7*-expressing neurons responsible for this rescue, GFP was positioned near the predicted fourth TM domain of UNC-7 (*unc-7SΔ::gfp*; Figure 4D). In other innexin constructs we found that placement of GFP near TM4 (deleting most of the carboxyl terminus) resulted in innexin::GFP proteins that did not localize to puncta but remained associated with the cell soma, making cell body identification possible (these studies and data not shown). An anti-GFP antibody was used to enhance detection (Figure 4D). As expected, UNC-7SΔ::GFP did not rescue locomotion, but allowed for identification of a number of head and tail neurons, including the locomotory command interneurons AVA and AVB (Table 3). Significantly, there was no evidence of motor neuron expression. Additionally, the *Punc-7S *promoter region was used in a translational fusion with the GFP reporter pPD95.67 (provided by A Fire), and this construct recapitulated the UNC-7SΔ::GFP expression pattern.

**Table 3:** Neurons identified by *Punc-7S *expression

Neuron	Gap junction partners*
AIB	AFD, DVB, DVC, RIG, RIS, RIV
AVA	ASn, DAn, VAn, SAB, PVC, RIM, URY, AVA, (DB5)
AVB	DBn, VBn, DVA, PVN, RIB, RID, SDQ, SIBV, AVB
AVE	RIM, **RMD**, **RME**, AVE
AVG	RIF
AVH	AVF, PHB, AVH
AVK	ADE, AQR, DVB, PVP, RIC, RIG, **SMB**, AVK
BDU	None described
CEPD	OLQ, RIH
FLP	AVD, RIH, FLP
PDE	PVC, PVM, VD9
RIA	None described
RIS	AIB, AVJ, RIM, SMD
RIVR	AIB, SDQ, **SMDD, SMDV**
RMDL/R	RMDD, **RMDV**
RMDVL/R	**AVE**, OLQ, **RMD**, RMDD, RMDV, **SAA**, **SMDV**, (muscle arms)
RMED	GLRD, IL1V, RIP, **RMEV**
RMEV	**AVE**, GLRV, IL1D, RIP, RMEL/R, **RMED**
SAAD	RMDD, **SMBD**
SIAD	RIB
SIAV	RIB
SIBD	AIM, RIB, SIBV
SMBD	**AVK**, RIB, **SAAD**
SMBV	**AVK**, SAAV
SMDD	RIB, RMDD, **RIS**
SMDV	RIB, **RIS**, **RMDV**, SMDV
I1	RIP, I1, (I2, I5)

*From White *et al*. [13]. GJ partners in bold are gap junction partners expressing the same INX. Gap junction partners in parentheses may form an occasional gap junction with the associated neuron.

Because UNC-7SΔ::GFP was not a rescuing construct, we also examined expression of the equivalent construct consisting of only native *unc-7 *sequences (*unc-7S*; Figure 3C, lacking GFP) using anti-UNC-7. As expected, this construct (minus F56B12) rescued forward but not backward locomotion, and UNC-7S was strongly expressed as puncta in the nerve ring and ventral nerve cord, but not the dorsal nerve cord (consistent with lack of DA and DB motor neuron expression). Together these studies suggest that *Punc-7S *drives expression of UNC-7S in a subset of neurons (including AVA and AVB, but not motor neurons) that can effect rescue of forward locomotion, and promoter sequences upstream of exon 2 drive additional expression of UNC-7S in motor neurons or possibly other neurons required to fully rescue all *unc-7 *locomotory defects.

### Other neuronal innexins are candidates for contributing to the locomotory nervous system

**Figure 5:**
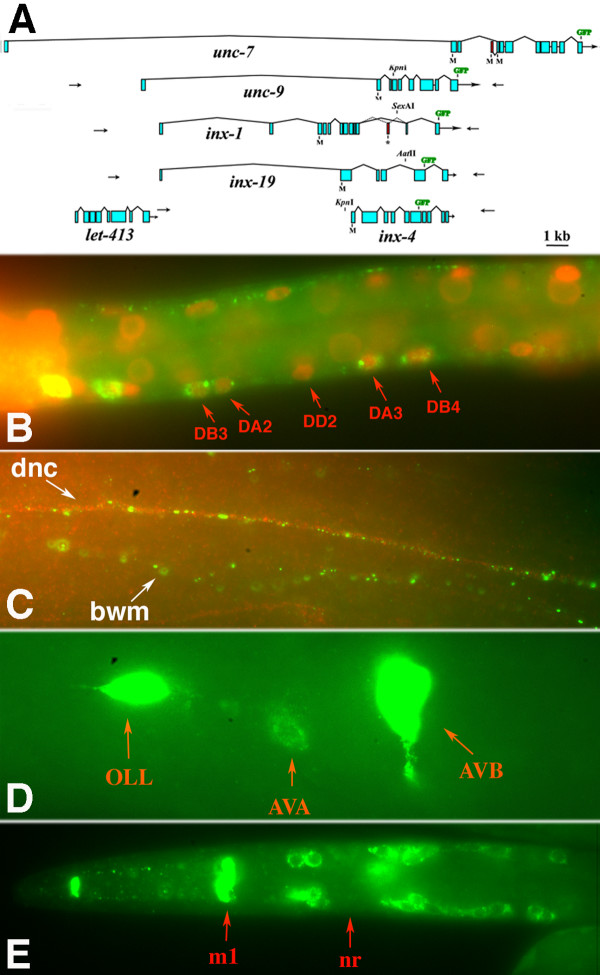
**Expression patterns of other neuronal innexins**. **(A) **Predicted gene structures of neuronal innexins. Arrows indicate primer binding sites used for PCR-amplified green fluorescent protein (GFP) constructs (see Materials and methods). **(B) **INX-1::GFP expression (green) in motor neurons; DAPI stained nuclei in red. **(C) **INX-1::GFP expression (green) in dorsal nerve cord (dnc) and body wall muscles (bwm); dorsal nerve cord is visualized with anti-UNC-33 antibody (red). **(D) **INX-19::GFP expression in AVA and AVB interneurons. **(E) **INX-4::GFP expression in sensory neurons and pharyngeal m1 muscle cell. Abbreviations: nr, nerve ring.

*unc-7 *is an unusually large *C. elegans *gene with a non-coding exon 1 followed by a large first intron. The predicted gene structures (including multiple isoforms) of some other worm innexins are similar; isolated cDNAs support this gene structure model for *unc-9*, *inx-1 *and *inx-19 *(WormBase), and a lack of coding sequence 7 kb upstream of the *inx-4 *translational start site made it another potential candidate. (*inx-19*, named by virtue of innexin sequence similarity, has been identified mutationally as *nsy-5*, shown to be critical for asymmetric fate determination in a pair of olfactory neurons in *C. elegans *[23].) We investigated the expression of these innexins and others [24] as potential candidates for interacting with UNC-7S in the locomotory nervous system. We used a long-range PCR strategy to generate GFP fusions representing *inx-1*, *inx-4*, *nsy-5*/*inx-19*, and *unc-9 *(Figure 5A); all four constructs were neuronally expressed to some extent (Figure 5B; Additional file 2). Characterization has not been exhaustive but some of the expression noted may be pertinent to locomotion.

INX-1::GFP is expressed in the nerve ring, ventral and dorsal nerve cords, and pharynx and body muscles (Figure 5B, C). Expression in DB and DA motor neurons can be seen in L1 stage larvae. Co-staining with anti-UNC-7 does not reveal any co-localization with INX-1::GFP (not shown), and expression of INX-1::GFP in *unc-7(e5) *mutants does not grossly affect its expression. INX-1::GFP appears to lie in a different focal plane from UNC-7 in the nerve cords (which may relate to expression in highly-coupled muscle arms that extend to the nerve cords). In larvae, NSY-5/INX-19::GFP is strongly expressed in OLL and AVB interneurons (Figure 5D), and weakly in a few other neurons, including AVA; more neurons express *nsy-5*/*inx-19::gfp *embryonically. Therefore, NSY-5/INX-19 could contribute to coordinated locomotion via expression in relevant command interneurons.

INX-4::GFP does not appear to be expressed in neurons implicated in locomotion, but is expressed in a number of ciliated neurons, pharyngeal m1 muscle cells, and the RIP neurons that form gap junctions with pharyngeal I1 neurons (Figure 5E). As such it is a candidate for potentially interacting with UNC-7S in the pharynx, and with NSY-5/INX-19 expressed in an overlapping set of sensory neurons [23].

UNC-9::GFP was broadly expressed throughout the nervous system in a pattern that greatly resembled UNC-7S, and we focused on potential interactions between UNC-7S and UNC-9, as described following.

### UNC-7 and UNC-9::GFP co-localize

Transformation rescue was used to assess how well UNC-9::GFP might represent native UNC-9 expression. First, the genomic region represented by the *unc-9::gfp *construct was shown to fully rescue *unc-9(fc16) *animals, indicating that this region is sufficient to encode a functional *unc-9 *product. GFP was positioned at the carboxyl terminus of UNC-9, without loss or interruption of UNC-9 sequence. The *unc-9::gfp *construct partially rescued uncoordinated locomotion in *unc-9(fc16) *– hermaphrodites exhibit wild-type forward movement interrupted with bouts of spastic kinking. Possibly the GFP moiety interferes with function to some extent.

**Figure 6:**
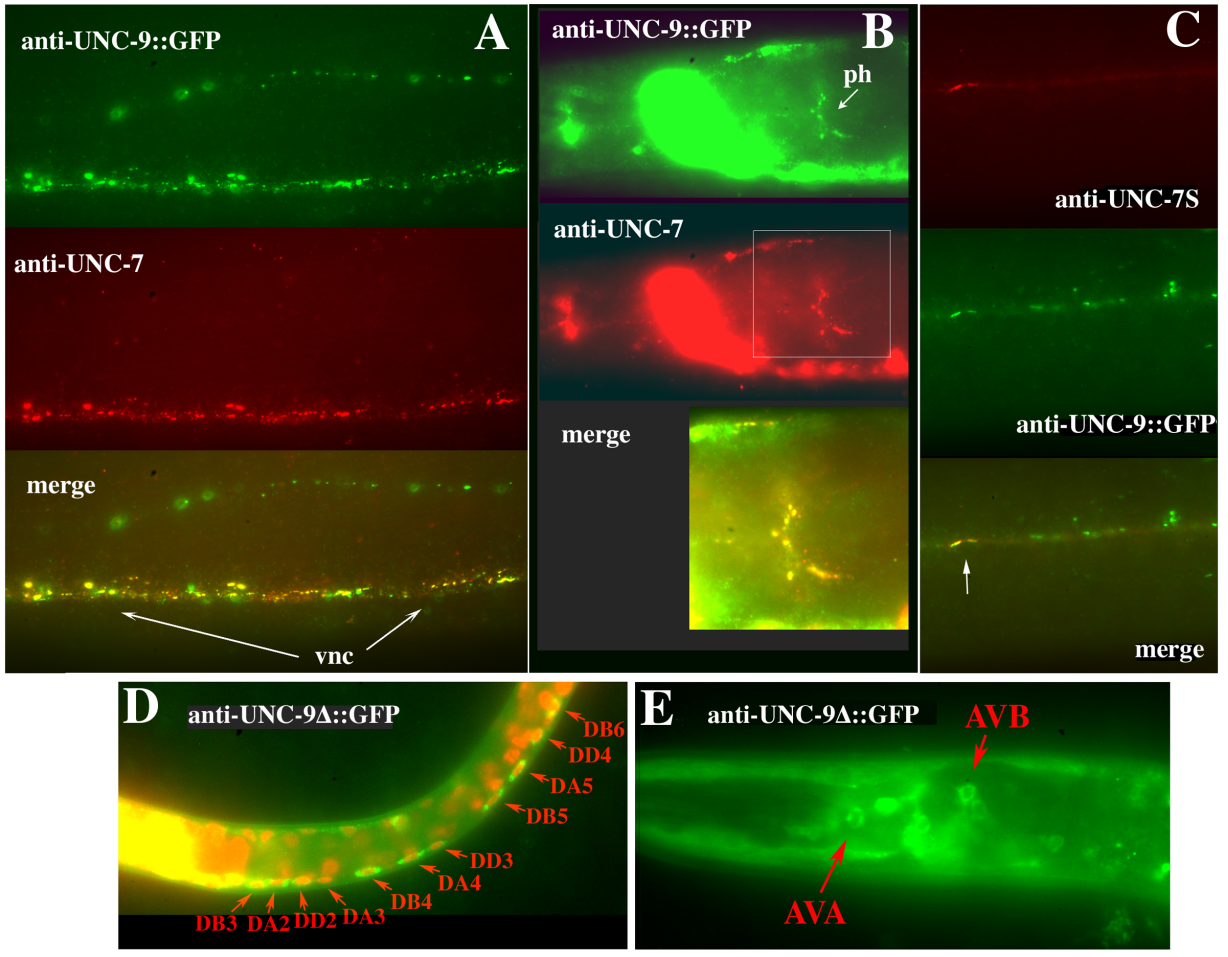
**UNC-7 and UNC-9::GFP co-localize**. **(A) **Section of ventral nerve cord (vnc) in a wild-type (N2) animal transformed with UNC-9::GFP. UNC-9::GFP signal enhanced with anti-GFP antibody (green); anti-UNC-7 (red); co-localization (yellow). UNC-9::GFP in body muscle seen in upper half of photo. **(B) **Co-localization in pharyngeal nerve ring (ph). **(C) **Co-localization (arrow) in an *unc-7(e5) *animal rescued for forward locomotion with *unc-7S *construct (Figure 3C, minus GFP). **(D) **UNC-9Δ::GFP expression in L1 stage, and **(E) **in head neurons of L4 stage.

Expression of *unc-9::gfp *greatly resembled that seen with anti-UNC-7 antibodies and *unc-7::gfp *constructs, and extensive co-localization was observed in the ventral and dorsal nerve cords and pharyngeal nervous system (Figure 6A, B). Additionally, UNC-9::GFP is expressed in body wall muscles to a much greater extent than UNC-7. Over-expression of *unc-9::gfp *elicits a strong kinker phenotype. Very high expression levels result in severe paralysis, with UNC-9::GFP detected in many puncta that do not co-localize with UNC-7. UNC-9 plays a role in coupling body muscle cells in *C. elegans *[25,26], and we speculate that paralysis could be due to over-expression in body muscles. Genetic mosaics of *unc-9 *were isolated in a manner identical to that used for *unc-7 *[9] to determine the focus of action of UNC-9. Seven animals were isolated that lost *unc-9(+) *in the P1 lineage giving rise to body muscle. These animals did not display an *unc-9 *phenotype, suggesting that *unc-9 *function is not required in muscle cells for coordinated locomotion (see Materials and methods).

*unc-7 *and *unc-9 *have identical Unc phenotypes. The co-localization of UNC-7 and UNC-9 suggested that these gene products interact. To test whether *unc-7 *and *unc-9 *might function in a common pathway, an *unc-9(fc16) daf-6(e1377) unc-7(e5) X *strain was constructed. *unc-9(fc16) *represents a probable null mutation [21]. *daf-6 *was used to facilitate construction and has no locomotory defects [27]. The phenotype of *unc-9 daf-6 unc-7 *mutants was no more severe than *unc-7 *alone, consistent with UNC-7 and UNC-9 acting in the same process.

### UNC-7S requires UNC-9 for localization

**Figure 7:**
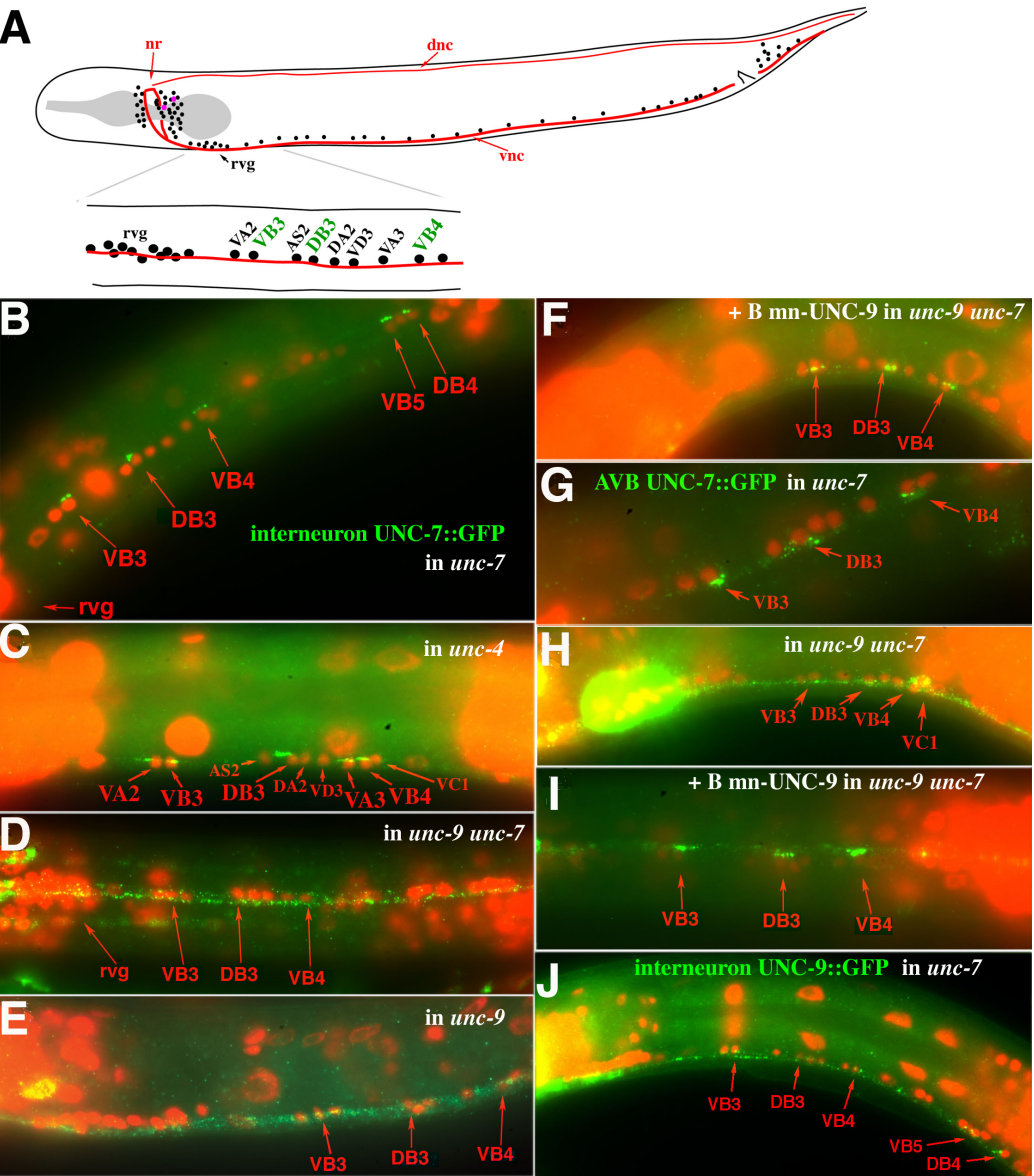
**UNC-7S::GFP expressed in AVB requires UNC-9 expression in B motor neurons for proper localization to AVB:B gap junctions**. **(A) **Diagram of anterior portion of ventral nerve cord (vnc) examined for expression of presumptive AVB:B motor neuron gap junctions. For clarity not all neuronal nuclei are represented; approximate positions of AVA and AVB (purple) posterior to the nerve ring (nr) are indicated. Motor neuron cell bodies lie along the vnc; D class motor neurons send processes dorsally to the dorsal nerve cord (dnc). The retrovesicular ganglion (rvg) is comprised of 20 neurons (including ten motor neurons) on the ventral side; the identity of neurons immediately posterior to the rvg can often be inferred by position, though some variation can occur [37]. **(B) ***unc-7S::gfp *construct expressed in *unc-7(e5)*. UNC-7S::GFP expression enhanced with anti-GFP antibody (green); DAPI-stained cell nuclei (red). **(C) **In *unc-4(e120) *UNC-7S::GFP additionally localizes to AVB:VA motor neuron gap junctions. **(D, E) **UNC-7S::GFP localization is more diffuse in *unc-9 daf-6 unc-7 *(D), and *unc-9 *single mutants (E). **(F) **Localization is rescued in *unc-9 daf-6 unc-7 *by co-expressing UNC-9 in B class motor neurons (*Pacr-5::unc-9)*. **(G-I) **UNC-7S::GFP expressed in AVB interneurons (*Psra-11::unc-7S::gfp*) in *unc-7(e5*) localizes to AVB:B gap junctions (G); localization is lost in *unc-9 daf-6 unc-7 *animals (H), but is rescued by UNC-9 expression in B motor neurons (*Pacr-5::unc-9*) (I). **(J) **UNC-9::GFP expressed in AVB (*Punc-7S::unc-9::gfp*) in *unc-7(e5) *animals fails to localize near B motor neuron cell bodies and is diffusely distributed along the ventral nerve cord.

Closer examination of UNC-7S expression resulting from rescue of forward locomotion in *e5 *animals by *unc-7S::gfp *or *unc-7S *revealed a distinctive pattern along the ventral nerve cord – small clusters of bright puncta were intermittently distributed and positioned near B class motor neuron nuclei visualized by DAPI staining (Figures 6C and 7B). From EM reconstruction it had been noted that AVB tended to form gap junctions with B motor neurons near cell bodies rather than processes, whereas AVA:A class motor neuron gap junctions were less restricted in placement [13]. Motor neuron cell bodies lie adjacent to the ventral hypodermal ridge, and the bundle of processes comprising the ventral nerve cord run alongside these positions [13]; UNC-7S::GFP puncta were often positioned slightly offset from where the ventral nerve cord appeared to lie, consistent with a cell body association. We hypothesized that UNC-7S::GFP may visualize gap junctions between AVB and its motor neuron targets. *unc-4 *mutations change the synaptic input of VA motor neurons to that of VB motor neurons [28]; in *unc-4 *mutants the UNC-7S puncta additionally localized near VA nuclei (Figure 7C) in support of this hypothesis. These puncta have been previously used as an assay for motor neuron connectivity, and a quantitative analysis of the distribution of UNC-7S puncta in wild-type animals showed a strong association with B class but not A class motor neuron cell bodies [29].

We postulated that UNC-7S might rescue forward locomotion by providing a gap junction subunit in AVB hemichannels that form channels with B class motor neurons. Analysis of the expression pattern from the *unc-7::Sgfp *construct showed no evidence of motor neuron expression; therefore, it appeared that either expression of UNC-7S::GFP in motor neurons is not required for AVB:B motor neuron gap junctions to form, or analysis of the *Punc-7S *promoter failed to detect low levels of motor neuron expression.

If UNC-7S expressed in AVB contributes to AVB:B motor neuron gap junctions, then UNC-9 might interact with UNC-7S in these junctions to form heteromeric or heterotypic channels important for coordinated movement. To examine co-localization of interneuron-expressed UNC-7S and total UNC-9::GFP, the *unc-7S *construct (Figure 3C, minus GFP) was used to rescue forward locomotion in *unc-7(e5) *mutants expressing UNC-9::GFP. UNC-7S co-localized completely with UNC-9::GFP in the ventral nerve cord, although, as expected, total UNC-9::GFP signal was more extensive (Figure 6C).

To attempt visualization of neuron cell bodies expressing *unc-9*, a second UNC-9 construct was made with GFP positioned near TM4; UNC-9Δ::GFP was detected in all motor neuron classes and AVA and AVB interneurons (Figure 6D, E), as well as body muscles and other unidentified neurons. With respect to UNC-7S co-localization, this indicated that UNC-9 could potentially contribute subunits to hemichannels in the motor neurons, in AVB, or in both, and UNC-9 may play a role in the formation of ectopic gap junctions noted in *unc-7(e5) *(between AVA and B class motor neurons). To further explore a potential relationship, expression from the *unc-7S::gfp *construct was examined in *unc-9(fc16) daf-6 unc-7(e5) *mutants, where UNC-7S::GFP failed to localize near B motor neuron cell bodies but instead was diffusely distributed as small puncta throughout the ventral nerve cord (Figure 7D). This pattern was recapitulated in *unc-9(fc16) *single mutants (Figure 7E), indicating that UNC-9 is required for the proper assembly of UNC-7S-containing AVB:B motor neuron gap junctions. It is possible that UNC-7S could require UNC-9 as a heteromeric co-assembly partner in AVB hemichannels, or as a heterotypic docking partner in B motor neuron hemichannels.

### UNC-9 motor neuron expression rescues UNC-7S localization

Heterologous promoters were used to refine where UNC-7S and UNC-9 are required for AVB:B motor neuron gap junction formation. The *Pacr-5 *promoter is expressed in B motor neurons but not AVB or AVA interneurons [30]. The *unc-7S::gfp *construct was introduced with *Pacr-5::unc-9 *into *unc-9(fc16) daf-6 unc-7(e5) *mutants. UNC-7S::GFP localized to presumptive AVB:B motor neuron gap junctions (Figure 7F), indicating that UNC-9 expressed in motor neurons is sufficient for this localization. It also suggests that in *unc-9 *mutants, the native UNC-7 isoforms expressed in motor neurons (as detected by previous analysis of total UNC-7 expression) are insufficient to properly localize AVB-expressed UNC-7S::GFP (that is, UNC-7 in motor neurons does not substitute for UNC-9 in localizing AVB-expressed UNC-7S).

To verify an AVB but not motor neuron requirement for UNC-7S, UNC-7S::GFP under control of the *sra-11 *promoter [31] was expressed in *unc-7(e5)*. *Psra-11::unc-7S::gfp *was detected in AVB, ALA, the pharyngeal neuron I4, an unidentified pair of neurons anterior to the nerve ring, and, in later larval stages, the VC motor neurons (but not A or B class motor neurons). GFP was seen in puncta near B class motor neuron cell bodies (Figure 7G), although these were generally not as pronounced as with *unc-7S::gfp*. When *Psra-11::unc-7S::gfp *was expressed in *unc-9 daf-6 unc-7*, puncta were not preferentially localized near B motor neurons (Figure 7H), but when co-injected with *Pacr-5::unc-9 *(B motor neurons) UNC-7S::GFP localization was restored (Figure 7I). These results further support the model that UNC-9 is sufficient in B motor neurons for gap junctions to form with UNC-7S in AVB.

We asked if UNC-9 expression in AVB might substitute for loss of UNC-7S and form gap junction channels with UNC-9 expressed in B motor neurons. *Punc-7S::unc-9::gfp *was expressed in *unc-7(e5) *mutants; UNC-9::GFP was broadly but weakly expressed as small puncta throughout the ventral nerve cord (Figure 7J), a pattern reminiscent of UNC-7S expression in *unc-9 unc-7 *double mutants (Figure 7D). Some of these puncta could potentially be associated with B motor neuron cell bodies – they appeared to be offset from other puncta expressed along the ventral nerve cord – but the intensity of their signal did not stand out compared to other puncta. If homotypic UNC-9 channels form between AVB and B motor neurons, they do not appear to lead to the establishment of electrical synapses as robust as those formed between UNC-7S and UNC-9 hemichannels. We conclude that there is a specific requirement for expression of the UNC-7S innexin in AVB to properly establish AVB:B gap junctions.

### UNC-9::GFP expressed in B motor neurons is mis-localized in *unc-7 *mutants

**Figure 8:**
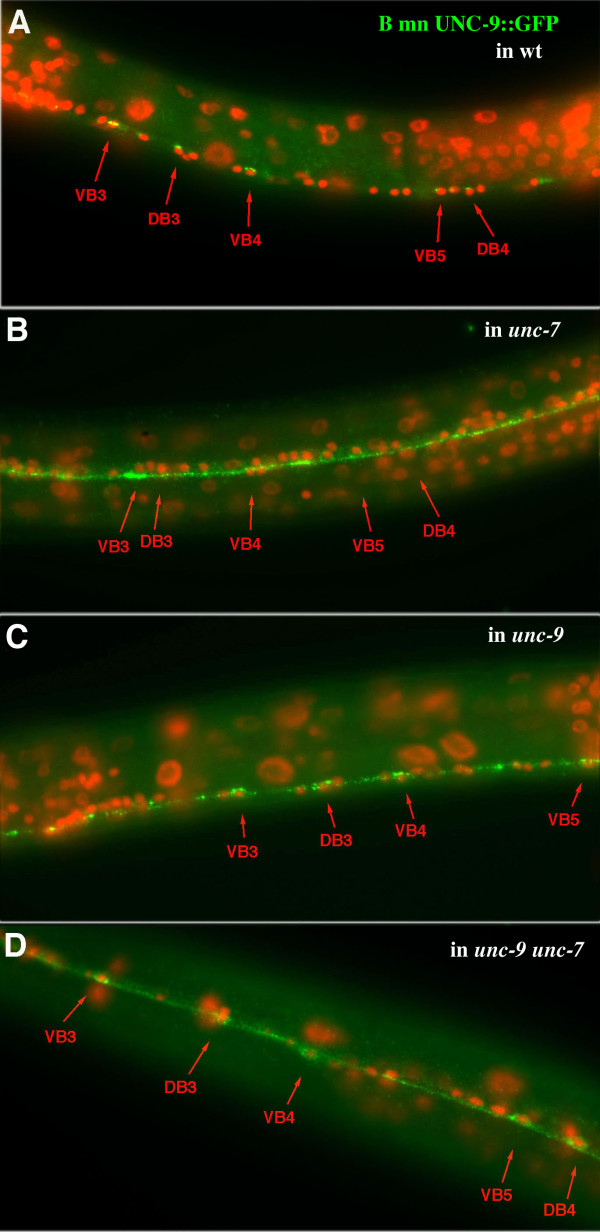
**UNC-9::GFP expessed in B motor neurons requires UNC-7 in AVB for proper localization to AVB:B gap junctions**. **(A) **In wild-type (wt; N2), UNC-9::GFP expressed in B motor neurons (*Pacr-5::unc-9::gfp*) visualizes puncta in the ventral nerve cord localized near B cell bodies. **(B) **UNC-9::GFP is expressed more uniformly in the ventral nerve cord in *unc-7(e5) *animals, and clusters of puncta near B cell bodies are not discernible. **(C) **In *unc-9(fc16)*, UNC-9::GFP puncta are more widely distributed, but the brightest puncta remain associated with B cell bodies. **(D) **In *unc-9 daf-6 unc-7 *animals, *Pacr-5::unc-9::gfp *is expressed more diffusely in cell bodies (few bright puncta).

Proper localization of UNC-7S in AVB requires UNC-9 expression in B motor neurons. Is there a reciprocal dependency for UNC-9 on UNC-7S? B motor neuron expression of *Pacr-5::unc-9::gfp *was examined in wild-type and various mutant backgrounds. In wild-type animals, *Pacr-5::unc-9::gfp *is expressed along the ventral nerve cord primarily as bright puncta situated near B motor neuron cell bodies (Figure 8A), a pattern mirroring that of UNC-7S expressed in AVB. In *unc-7 *mutants, bright *Pacr-5::unc-9::gfp *puncta are scattered throughout the ventral nerve cord and are not particularly concentrated near B motor neuron cell bodies (Figure 8B), suggesting that UNC-7S is required for the wild-type localization of UNC-9::GFP to AVB:B gap junctions.

In *unc-9 *mutants, the brightest UNC-9::GFP puncta are still localized near B cell bodies, but more puncta are distributed elsewhere along the cord compared to wild-type (Figure 8C). We can only speculate as to why there is a wider distribution of UNC-9::GFP puncta in an *unc-9 *background. Our genomic *unc-9::gfp *construct was found to only partially rescue *unc-9 *mutants. Perhaps in wild-type animals, association of native UNC-9 with UNC-9::GFP in the same hemichannels allows for stricter control of UNC-9::GFP distribution than in *unc-9 *mutants.

In *unc-9 unc-7 *double mutants, the UNC-9::GFP signal is qualitatively different – few bright punta arise, expression is more diffuse, and the GFP signal often concentrates in the cell soma of some B motor neurons, allowing for their visualization.

### UNC-7 and UNC-9 form channels in *Xenopus *oocytes

**Figure 9:**
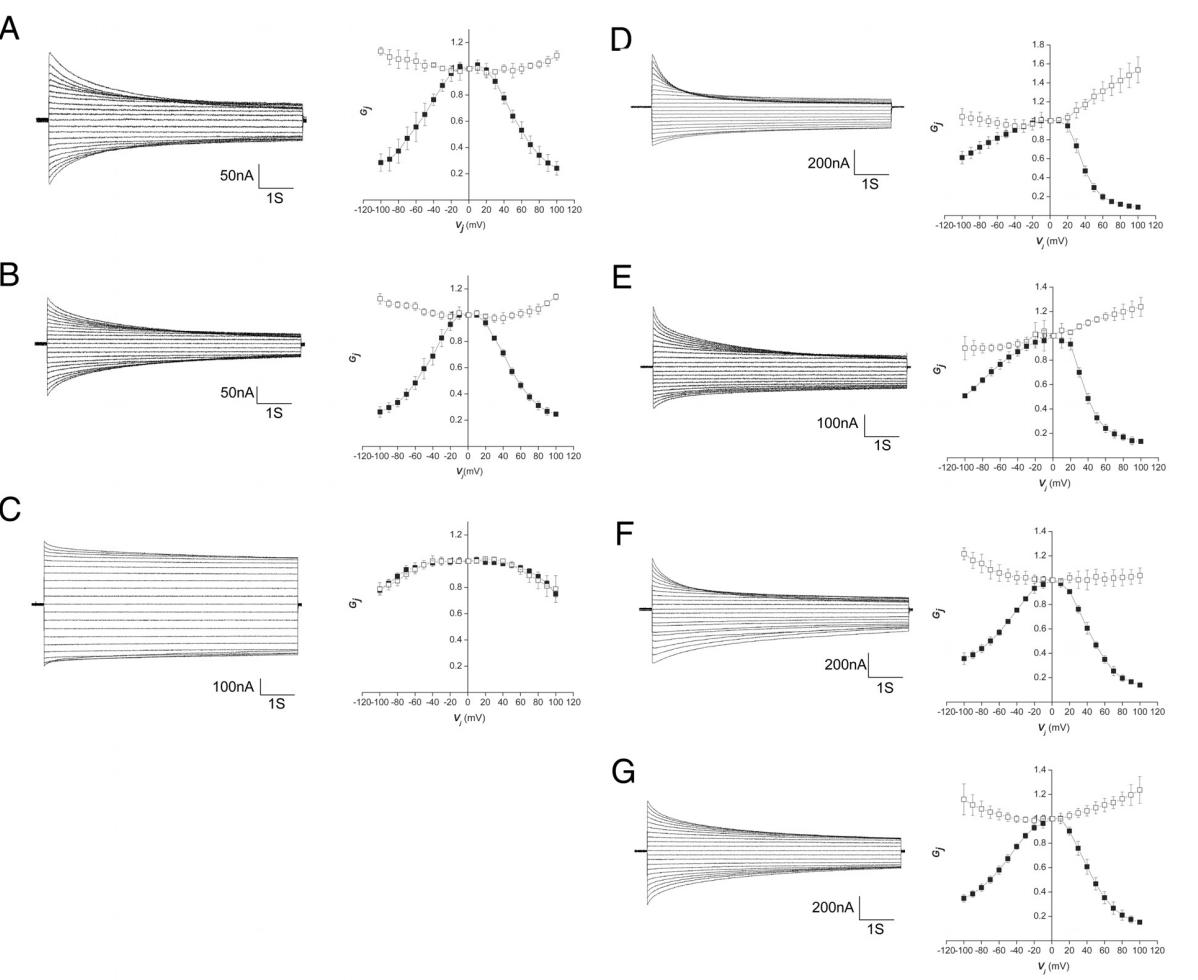
**Functional analysis of UNC-7 and UNC-9 in *Xenopus *oocytes**. Left: superimposed traces of junctional currents evoked by 10 mV voltage steps from -100 to +100 mV in one of a pair of *Xenopus *oocytes injected with cRNAs for the UNC proteins indicated below. Right: graphs of conductance/transjunctional voltage (*G*_j_/*V*_j_) relations for initial conductance (*G*j_0_, open squares) and steady state conductance measured at the end of the 15 s pulse (*G*_j∞_, closed squares) averaged from four to five cell pairs. *G*_j0 _values are normalized to G_j0 _at *V*_j _= 0, while *G*_j∞ _values are normalized to *G*_j0 _at the same voltage (*G*_j∞_/*G*_j0_). Error bars represent standard deviation, n = 5. Results shown are for the following pairings, where relative polarity of Vj is defined with respect to the innexins on the right in each pair: **(A) **UNC-7S/UNC-7S; **(B) **UNC-7L/UNC-7L; **(C) **UNC-9/UNC-9; **(D) **UNC-7S/UNC-9; **(E) **UNC-7L/UNC-9; **(F) **UNC-7S/UNC-7L; **(G) **UNC-7S/UNC-7L+UNC-9, where equal quantities of UNC-7L and UNC-9 cRNA were co-injected into the right cell.

To further explore the relationship between UNC-7 isoforms and UNC-9, studies in the *Xenopus *oocyte exogenous expression system were undertaken. This system has consistently reconstituted the *in vivo *behavior of connexins in the absence of endogenous background and has been used to express innexins effectively [8,32,33]. UNC-7S, UNC-7L and UNC-9 cDNAs were cloned into pCS2+ for *in vitro *preparation of mRNA and oocyte injection. Typically, all three innexins were found to form homotypic channels with symmetrical gating properties in response to transjunctional voltage (V_j_) (Figure 9A–C). The decay in junctional currents, even at high V_j_, is relatively slow, taking approximately 15 s to reach steady state. The initial conductances (G_j0_) for both UNC-7 isoforms increase slightly with V_j _of either polarity. This is in contrast to UNC-9, where G_j0 _decreases with V_j _of either polarity, indicating a rapid gating event that was not resolved by the current clamp. The slower gating response of these channels can be followed by plotting the steady state conductance (G_j∞_) normalized to G_j0 _at the same voltage. Both UNC-7 isoforms show symmetrical gating profiles that can be fit by Boltzmann relations (Figure 9A, B) with parameters that are indistinguishable (Additional file 3). By contrast, UNC-9 showed much less sensitivity to V_j_, with G_j∞ _showing a similar dependence on V_j _as G_j0 _showed (Figure 9C). Since G_j∞ _did not approach a minimum value within the V_j _range studied, the data could not be fit reliably to a Boltzmann relation.

All UNC isoforms were also tested for the ability to form heterotypic channels, given the indications from localization studies that AVB:B motor neuron gap junctions are likely heterotypic. UNC-7S and UNC-7L each formed heterotypic channels with UNC-9, with asymmetric gating profiles that again were essentially indistinguishable from one another (Figure 9D, E). Given that the gating profile when the UNC-7S or L side is relatively negative (UNC-9 positive) is similar to that of homotypic UNC-7, and when UNC-9 is relatively negative the limited gating response resembles UNC-9 homotypic, we conclude that these members of the innexin family close the series gate on the negative side (this is the reverse of most, but not all, connexins [7]). However, it did appear that the hemichannels of all of the innexins studied here showed a higher sensitity to V_j _in the heterotypic conformation compared to the homotypic, as reflected in lower G_min_s and V_0_, and higher A values (Additional file 3). The asymmetry of the V_j _response of these UNC-7:UNC-9 heterotypic channels was not restricted to the steady state response of these cells, but also was seen in the rectifying nature of the initial conductance that increases as the UNC-9 cell becomes more positive. Such rectifying behavior has been reported for some connexins, and has been shown to arise from the pairing of two hemichannels with different ion selectivities [34]. Based on these previous modeling studies, we would predict that UNC-9 channels are more cation selective than either of the UNC-7 isoforms.

The UNC-7 isoforms were also found to form heterotypic channels with one another (Figure 9F). These channels were more symmetric, but did show some differences, particularly in terms of the G_min _for each side and the rectification of G_j0_. We also tested more complex pairings by co-expressing multiple innexins within one cell. UNC-7S was expressed in one oocyte and equal amounts of UNC-7L and UNC-9 RNA were injected in the other. The channels showed a V_j _gating profile that was intermediate between UNC-7S:UNC-9 (Figure 9D) and UNC-7S:UNC-7L channels (Figure 9F) in that the G_j0 _response was intermediate between those of UNC-7S:UNC-9 and UNC-7S:UNC-7L pairings, while the G_j∞ _was similar to the UNC-7S:UNC-7L pairing (see Boltzmann parameters in Additional file 3). This could be interpreted as UNC-7L and UNC-9 forming heteromeric channels, with ion selectivity properties that are influenced by both isoforms (as this dictates the rectification of G_j0_), but V_j _gating properties dominated by UNC-7L (as this dictates the shape of the G_j∞ _plot). Alternatively, the data could also be consistent with a mixture of heterotypic pairings of UNC-7S hemichannels with homomeric UNC-7L or UNC-9 hemichannels, where pairings with UNC-7L are favored (explaining the dominant influence of UNC-7L on G_j∞_). Even in the case of G_j0_, the response is somewhat more similar to an UNC-7 response than UNC-9, and thus is not inconsistent with this model.

The marked similarity between the voltage gating behavior of UNC-7S and L could indicate that the amino terminus has little effect on voltage gating of these channels, in stark contrast to what has been found for connexins (summarized in [35]); alternatively, it is possible that both *unc-7 *expression constructs might utilize the internal start site (M121) and similarly produce UNC-7SR. To test this possibility, UNC-7L(M121L) mRNA was transcribed from pCS2+. This mRNA, which should no longer generate the UNC-7SR product, failed to produce intercellular currents in oocytes, suggesting that the previously observed UNC-7L currents may reflect inclusion of UNC-7SR translation products. However, the asymmetry seen in the UNC-7S:UNC-7L pairings (Figure 9F) may indicate that some of the full length translation product, while not capable of forming functional channels alone, could possibly combine with the shorter product and modify its gating. Results from SDS-PAGE analysis of *in vitro *translation products derived from UNC-7 mRNAs (in rabbit reticulocyte lysates) are consistent with this interpretation (Additional file 4).

### UNC-7L and UNC-7S fail to heteromerize with UNC-9::GFP

**Figure 10:**
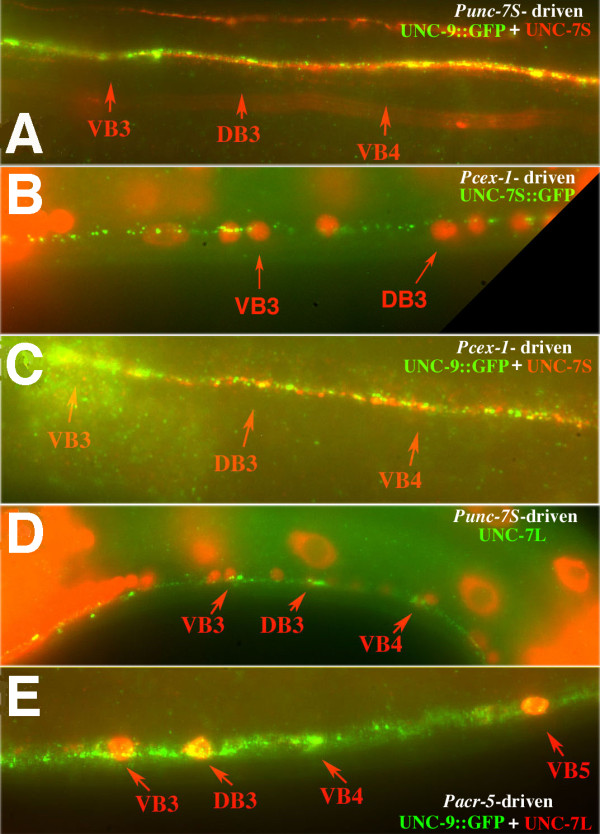
**UNC-9 and UNC-7 fail to heteromerize**. **(A) **Co-expression in interneurons of UNC-7S (red) and UNC-9::GFP (*Punc-7S::unc-9::gfp*; green) in *unc-7(e5) *shows little co-localization (yellow). (For clarity DAPI staining is not shown; positions of B motor neuron nuclei are indicated.) UNC-7S does not concentrate in puncta near B cell bodies. **(B) ***Pcex-1::unc-7S::gfp *(AVA, AVD interneurons) rescues forward locomotion but does not concentrate in puncta near B cell bodies. **(C) **Co-expression of *Pcex-1::unc-7S *(red) and *Pcex-1::unc-9::gfp *(green) shows little co-localization in an *unc-7(e5) *background. **(D) **UNC-7L expressed in interneurons (*Punc-7S::unc-7L [M121L]*) rescues forward locomotion in *unc-7(e5) *and localizes to puncta near B motor neuron cell bodies. **(E) **UNC-7L expressed in B motor neurons (*Pacr-5::unc-7L [M121L]*) in *unc-9 daf-6 unc-7 *does not localize to puncta but remains associated with cell bodies (red); co-expressed UNC-9::GFP (*Pacr-5::unc-9::gfp*; green) is distributed throughout the motor neuron processes.

Studies in *Xenopus *oocyte pairs could not provide a definitive resolution of whether UNC-7 and UNC-9 isoforms heteromerize, although it is clear that heterotypic interactions between all three isoforms studied here can and do form, even if some may be favored. We investigated potential heteromerization *in vivo*. UNC-7S and UNC-9::GFP were co-expressed in different neuron subsets represented by *Punc-7S *or *Pcex-1 *(*unc-7S *+ *Punc-7S::unc-9::gfp*; *Pcex-1::unc-7s *+ *Pcex-1::unc-9::gfp*) in *e5 *animals (Figure 10A, C). The *Pcex-1 *promoter fragment we utilized is strongly expressed in AVA, AVD, RIM and 2 tail neurons but not AVB, and *Pcex-1::unc-7S *puncta are distributed throughout the ventral nerve cord (Figure 10B). In both cases very little co-localization between UNC-7S and UNC-9::GFP was seen; furthermore, UNC-7S expressed in AVB from its native promoter failed to form easily discernible AVB:B motor neuron puncta normally associated with its expression (Figure 10A).

When expressed under control of the *Punc-7S *promoter, UNC-7L (*Punc-7S::unc-7L [M121L]*) rescued *unc-7(e5) *forward locomotion; though much of the signal remained associated with cell bodies, some puncta were seen localized to AVB:B motor neuron gap junctions (Figure 10D). We asked if it was possible for UNC-7L to associate with UNC-9 in B class motor neurons. UNC-9::GFP and UNC-7L were co-expressed (*Pacr-5::unc-9::gfp *and *Pacr-5::unc-7L [M121L]*) in *unc-9 daf-6 unc-7 *animals. UNC-9::GFP was widely expressed in the ventral nerve cord as puncta, but UNC-7L was principally associated with cell bodies, and no co-localization was observed (Figure 10E). It therefore appears unlikely that UNC-9 heteromerizes with either UNC-7S or L isoforms. This result helps in the interpretation of the *Xenopus *oocyte studies, which now would suggest that UNC-7S in one cell would favor pairing heterotypically with UNC-7L over UNC-9 homomeric hemichannels expressed in the apposed cell. If this preferential pairing also worked in the opposite direction (that is, UNC-9 prefers to pair with UNC-9 over UNC-7 channels) this could explain why mis-expression or over-expression of UNC-9 in AVB (or other neurons) might interfere with UNC-7S localization to AVB:B motor neuron gap junctions. UNC-9 expressed in AVB cells might be expected to compete with, and displace, endogenous UNC-7S from these gap junctions.

**Figure 11:**
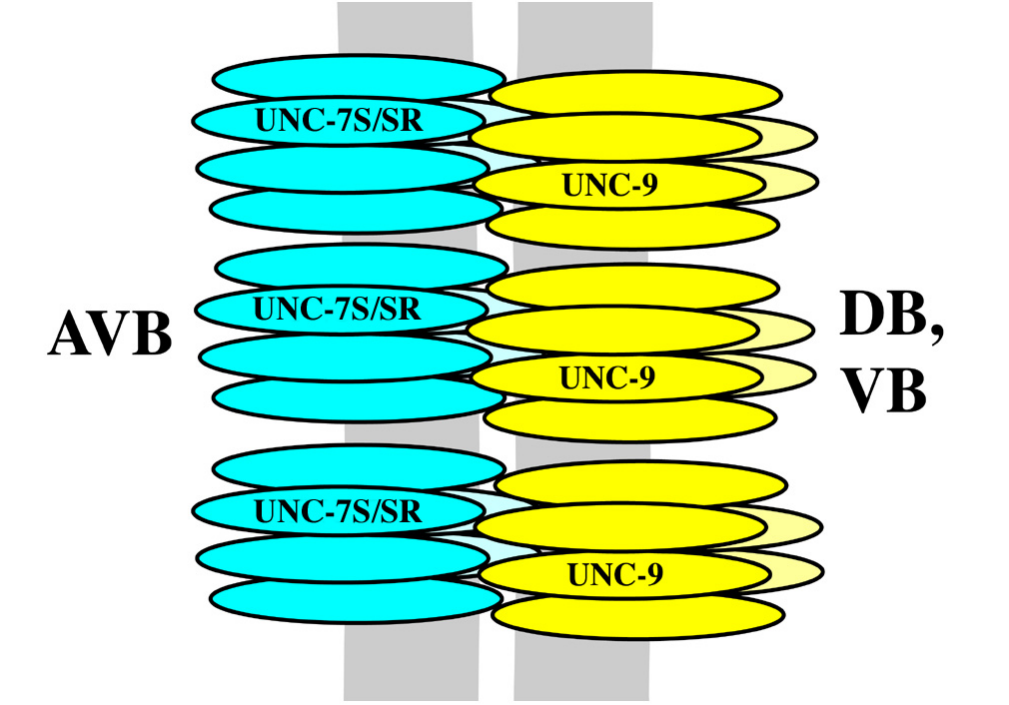
**Model for composition of heterotypic AVB: B motor neuron gap junction channels**.

These and previous results lead to the simple model that AVB:B motor neuron gap junction channels are homomeric and heterotypic, with hemichannels composed solely of UNC-7S provided by AVB and hemichannels composed solely of UNC-9 provided by the B motor neurons (Figure 11).

### Function of AVB:B motor neuron gap junctions

Rescue of forward locomotion in *unc-7 *mutants coincided with localization of UNC-7S to AVB:B motor neuron gap junctions; an attractive model emerged that UNC-7S in AVB might rescue forward locomotion by docking with UNC-9 in B motor neurons, restoring functional gap junctions. *Psra-11::unc-7S::gfp *expressed in AVB did not rescue forward locomotion in *e5 *animals; the *Pcex-1::unc-7s:gfp *construct, expressed in AVA and AVD but not AVB, did, however, rescue *e5 *forward locomotion. Therefore, one common feature of heterologous promoter::UNC-7S fusions that rescued forward locomotion was expression in AVA. AVA interneurons are implicated primarily in reverse rather than forward locomotion. Could undetectably low levels of UNC-7S expression driven by heterologous promoters in AVB account for rescue? To address this, genetic mosaic analysis of rescue of forward locomotion by the *unc-7S *construct was carried out. Briefly, we screened for mosaic animals that had lost the rescuing array somewhere in the AB lineage (giving rise to virtually all the interneurons and motor neurons implicated in locomotion) but still showed good forward movement. Results were consistent with an AVA requirement for UNC-7S expression (Additional file 5), suggesting that the formation of AVB:B gap junctions alone is insufficient to rescue forward movement. Rescue of forward locomotion through AVA expression of UNC-7S may indicate some underlying plasticity to the neural circuitry instructing locomotion.

## Discussion

### UNC-7 and UNC-9 interactions

A major motivation for characterizing *unc-7 *and *unc-9 *was to investigate how specificity of gap junction formation between neurons might be achieved. Although numerous studies have provided insights into factors impacting neural process migration, there is little understanding of how neurons make their final target selection. Understanding how electrical synapses are established may relate to the general question of how neurons recognize their eventual targets.

In the ventral nerve cord of *C. elegans*, neurons of the locomotory nervous system form gap junctions with some neighbors but not others; the mechanisms regulating recognition and formation of gap junctions between synaptic partners are unknown. From numerous studies in the *Xenopus *paired oocyte system and various cell culture systems, a reasonable assumption might be that if two cells express the same connexin or innexin and lie in close membrane apposition, gap junctions will probably be established between them; this assumption has not been investigated rigorously *in vivo*. Reconstruction of the *C. elegans *nervous system by EM serial section showed that 92 of 104 motor neuron classes establish gap junctions [13]. This assessment excludes any gap junctions that might be developmentally specific (for example, see [23]). Although there are 25 innexin genes, only a subset of these are neuronally expressed. We sought to investigate if the scale of electrical synaptic specificity seen in the *C. elegans *nervous system could be explained through a model whereby gap junction formation between neighbors is restricted by the identity of expressed innexins.

Characterization of the *unc-7 *locus led to identification of multiple isoforms, offering the potential that isoforms could play different roles in determining synaptic specificity. Because *unc-7 *and *unc-9 *mutants are indistinguishable, it has long been thought that these genes may interact. The phenotype of *unc-9 unc-7 *double mutants is no more severe than either single mutant phenotype, consistent with the co-localization of UNC-9::GFP and UNC-7S. Experiments to dissect the nature of UNC-7 and UNC-9 interactions were governed by the complex nature of gap junctions, that is, a gap junction plaque may consist of hundreds of single channels, and co-localization of innexins might represent: individual homomeric channels coalesced in the same gap junction plaque; localization to different homomeric hemichannels in the same heterotypic channel; or heteromeric subunits in a single hemichannel. From the widespread and overlapping expression patterns of UNC-7S and UNC-9, it appeared likely that both of these innexins contribute subunits to most of the gap junctions in the locomotory nervous system.

Restricted expression of UNC-7S from the *Punc-7S *promoter allowed us to recognize AVB:B motor neuron gap junctions in live animals and has served as a useful tool for examining changes in synaptic connectivity [29]. Our present analysis leads us to propose a model that these gap junctions are heterotypic for UNC-7S and UNC-9. Phenotypic rescue is commonly used as a measure of wild-type function, and expression of UNC-7S on the AVB side of these junctions was coincident with rescue of forward locomotion. However, use of heterologous promoters and genetic mosaic analysis indicated that rescue can be effected by UNC-7S expression in AVA, and, therefore, locomotion rescue is not a reliable measure of functional reconstitution of AVB:B gap junctions; other means had to be employed to assess the innexin composition of these channels.

Using *unc-9 *mutants and heterologous promoters, we showed that expression of UNC-9 in B class motor neurons is necessary to rescue the localization of UNC-7S expressed in AVB. A reciprocal dependence for UNC-9::GFP expressed in B motor neurons on AVB-expressed UNC-7S was found. This was somewhat surprising given that both UNC-7S and UNC-9 can form homomeric channels in *Xenopus *oocytes. Possibly in the respective mutant backgrounds homomeric channels may form but are not recognizable by our methods because they do not particularly stand out as brighter puncta offset from the ventral nerve cord near B cell bodies. If such is the case, it would suggest that the strength of electrical synapse (size and brightness of punctum) may be dependent on the proper composition and function of the gap junction channels formed between AVB and B motor neurons; little is known about what factors may regulate the size or positioning (cell soma or cell process) of gap junction plaques.

Although we found evidence that UNC-9 and UNC-7S are both expressed in AVB and B motor neurons, co-expression of UNC-7 isoforms and UNC-9 in the same cells showed little evidence of co-localization, and in *Xenopus *oocytes showed little evidence of association, suggesting that these innexins do not heteromerize in the same hemichannel. We therefore favor the model as depicted in Figure 11. This does not rule out a possible contribution of other innexins to these channels, and candidates include INX-19::GFP in AVA and AVB and INX-1::GFP in motor neurons. However, UNC-7S::GFP was found to properly localize to AVB:B motor neuron gap junction plaques in *nsy-5/inx-19(tm1896) *loss-of-function mutant animals, and although INX-1 co-localizes extensively with UNC-9::GFP in body wall muscles, it does not co-localize with UNC-7::GFP or UNC-9::GFP in the nerve cords (data not shown). Recently, it was shown that the *Drosophila *innexins 2 and 3 interact through their carboxyl termini *in vitro *and *in vivo *[36]; likewise, our GFP constructs suggest that the carboxyl terminus of innexins is necessary for localization into plaques, possibly due to an inability to oligomerize. Co-localization or binding studies would be useful to show whether other innexins might heteromerize with UNC-7S or UNC-9 and possibly contribute to AVB:B gap junctions.

Our analysis of gap junction formation focussed primarily on later larval stages and adults. At this stage animals are large enough such that the cell bodies of motor neurons in the ventral nerve cord are well spaced, making it less ambiguous in deciding if presumptive gap junction puncta are associated with a particular motor neuron. However, the locomotory nervous system undergoes significant changes late in the first larval stage, involving the wiring of emergent and the rewiring of extant motor neurons [37]. Motor neurons at this stage appear to express at least four innexins, including *inx-3 *[38], *unc-7*, *unc-9*, and *inx-1 *(these studies). The relationship, if any, between these innexins and the rewiring involved is unknown. Understanding the relationship between UNC-7 and UNC-9 in establishing AVB:B motor neuron gap junctions found in later larval stages will provide a context for examining earlier developmental formation of electrical synapses.

### Gap junction function

The heterotypic relationship between UNC-7S and UNC-9 can explain why *unc-7 *and *unc-9 *mutants share identical phenotypes; however, it is still uncertain which of the gap junctions they form are critical for locomotion. Because over- or mis-expression of UNC-7S driven by *Punc-7S *in interneurons rescues forward locomotion in *e5 *animals, it appears that any intra-class motor neuron junctions to which UNC-7 contributes may not be crucial for locomotion, or UNC-9 homotypic channels might compensate for the loss of UNC-7 in motor neurons. It is of interest to note that gap junctions between motor neurons have not been observed in the highly similar *Ascaris *locomotory nervous system [39,40].

It was surprising that re-establishment of AVB:B motor neuron gap junctions was not implicated in the rescue of *unc-7(e5) *forward locomotion by *unc-7S*. Ablation of command interneurons by restricted expression of the human caspase ICE showed that even in the absence of interneuron input, worms can still move in a sinusoidal wave in a limited manner [41]. A model has been proposed that control of locomotion in *C. elegans *can be regarded as a distributed bistable switch regulating the time durations worms move forward or backward [41,42]. In such a model AVB:B gap junctions could play a role in maintaining the duration of forward locomotion or restricting the duration of backward locomotion; that is, AVB might be instructive to B class motor neurons or receptive to them. Here a comparison to *Ascaris *is of interest as well. Whereas in *C. elegans *forward locomotion is effected by a backwards propagating wave, *Ascaris *moves forward via forward-propagating waves [43]. Although the classes and arrangement of motor neurons in both species are virtually identical, there may be underlying changes in interneuron function that contribute to this difference. AVB gap junctions with B motor neurons may reflect at least part of this difference.

How the heterotypic nature of AVB:B motor neuron gap junction channels influences their function is unknown, but we found that in *Xenopus *oocytes, UNC-7S:UNC-9 heterotypic channels are rectifying both in terms of gating and instantaneous current flow, a property not found for either homomeric channel. Without more detailed analyses of the properties of the AVB:B motor neuron synapse *in vivo *it is difficult to divine the potential significance of this rectifying behavior. Since gating is only seen when the UNC-7 cell is relatively hyperpolarized compared to the UNC-9 cell, this would ensure that the channels do not close when depolarizing impulses are passed from AVB to B motor neurons. However, the time constant of the gating of these channels is so slow that it is unlikely to be significant in the time frame of a neuronal impulse. The rectification of G_j0 _would appear to favor conduction of depolarizing potentials from the motor neuron to AVB, rather than the reverse, but again the degree of rectification is minimal enough that it may not play a major role physiologically.

### Electrical synapse specificity

Most motor neurons in the locomotory nervous system appear to express both UNC-7 and UNC-9. Examination of innexin expression patterns ([24] and these studies) provided only a small number of other candidates that might contribute to specificity in the locomotory nervous system. Although the *unc-7 *locus encodes three isoforms that differ in their amino termini, two of these appear functionally equivalent, and the ability of UNC-7L to contribute to junctions is still uncertain. (It is possible that UNC-7L requires processing or association with another innexin to be incorporated into junctions, or has another function altogether.) Therefore, the simple model that gap junction synaptic specificity is determined strictly by differential expression of innexins unique to particular neuron classes seems untenable. Questions remain concerning why neurons that apparently express similar innexins (the different motor neuron classes) rarely form gap junctions with one another, if gap junctions are subject to constant formation and reformation in the nervous system, and why there appears to be a consistency in subcellular localization (soma versus process) of certain gap junctions.

## Conclusion

How synaptic specificity is achieved is poorly understood. Innexins UNC-7 and UNC-9 contribute to a large number of electrical synapses in the *C. elegans *locomotory nervous system. Examination of specific gap junctions between AVB interneurons and B class motor neurons indicated that these junctions are likely composed of heterotypic UNC-7S:UNC-9 gap junction channels, and the formation of these junctions specifically requires UNC-7S expression in AVB and UNC-9 expression in B class motor neurons. Co-expression of UNC-7 and UNC-9 in the same cells does not lead to heteromerization, and it is likely that most of the co-localization seen between these innexins represents heterotypic channels. Although interactions between UNC-7S and UNC-9 are critical for effecting electrical synaptic specificity, differential innexin expression is inadequate to account for the entirety of specificity observed among the interneurons and motor neurons involved in locomotion, and other factors must be involved. The ability to recognize specific electrical synapses may allow investigation of the factors determining how neurons recognize and establish gap junctions with their synaptic partners.

## Materials and methods

### Strains

Worms were cultured by standard techniques [44]. Bristol strain N2 was used as wild type. Mutant strains of *unc-7 X *included: CB5 *unc-7(e5)*, CB42 *unc-7(e42)*, CB65 *unc-7(e65)*, CB133 *unc-7(e133)*, CB139 *unc-7(e139)*, HH30 *unc-7(hs9)*, HH34 *unc-7(hs10)*, SP1376 *lin-2(e1309) unc-7(mn382)*, SP1379 *lin-2 unc-7(mn383)*, SP1380 *unc-7(mn384)*, and SP1396 *unc-7(mn409)*. Other strains used included CB1377 *daf-6(e1377) X *and CW129 *unc-9(fc16) X*. For transformation rescue, SP1531 *ncl-1(e1865) unc-36(e251) III *was sometimes used; the *ncl-1 *gene was incidental in these cases. Further information on transgenic strains provided on request.

### Laser ablation

A Bull's-Eye dye laser (Fryer, Huntley, IL, USA) excited by a VSL-337 nitrogen laser (Laser Science, Inc., Newton, MA, USA) was used on a Nikon Microphot FXA microscope to ablate AVA cells in L1 animals (for method, see [45]).

### Antibody production

Anti-UNC-7 antibodies were generated against the carboxyl-terminal 88 amino acids of UNC-7 (S435-D522), and affinity-purified to GST::UNC-7 covalently cross-linked to glutathione beads). Injections and antisera collection were performed by Quality Controlled Biochemicals, Inc. (Hopkinton, MA, USA). Anti-Unc-7 antibodies were used at a 1:40 dilution.

### 5' RLM-RACE

To identify the *unc-7S *transcript, a cDNA library (gift of L Bell) synthesized using the FirstChoice RLM-RACE kit (Ambion, Austin, TX, USA) was used for RNA ligase-mediated (RLM)-RACE amplification with *unc-7 *specific nested primers (5'-CAGTATGTGTTCTGTACC-3' and 5'-CATTGCAGGTCCGAACGC-3') according to the recommended protocol. The 5' end of *unc-7S *was identified as an SL1-spliced product with 94 nucleotides found in intron 3 (nucleotides 12,452-12,359) serving as exon 1. The predicted amino terminus sequence upstream of the canonical innexin start Met (5'-**MYHSKQANTKYIKK**AQKLLDGSHQLRIDSHHVGSAGHGAGQGHGHKKEFGPAMet) includes 14 amino acids unique to UNC-7S (in bold).

### *unc-7 *DNA constructs and phenotypic analysis

Most *unc-7 *genomic constructs were made using subcloned fragments derived from cosmids F09B12 and R07D5. The *unc-7S::gfp *construct (Figure 3C) begins at the *Sal*I site in exon 2 (nucleotide 14,412 of R07D5) and extends to a blunt-ended *Bam*HI site (nucleotide 5,789). The GFP sequence (GFP [afmx)]; Affymax Research Institute, Palo Alto, CA, USA) including a stop site was inserted into the *Sal*I site in exon 11 (nucleotides 8,828–8,833), resulting in deletion of 17 amino acids of the UNC-7 carboxyl terminus. This construct rescued forward locomotion when micro-injected at DNA concentrations ranging from 1–75 ng/μl. Derivatives of this basic construct included removal of the alternative exon 4 by deleting an *Nhe*I/*Apa*I fragment (nucleotides 12,259–12,543; Figure 3D); replacement of the genomic region covering exons 2–6 with the corresponding cDNA sequence (*Sal*I to *Nco*I; nucleotides 14,412–11,736; Figure 3E) plus additional upstream sequence to allow for recombination with F56B12 (to the *Xho*I site at nucleotide 15,574); and introduction of a Met to Leu mutation (M121L, ATG to CTG) by PCR amplification with primers that included the sequence change (nucleotides 11,968–11,970; Figure 3F). A fully-rescuing *unc-7 *construct (Figure 3B) extended to the *Xho*I site (nucleotide 15,574) and included a frameshift generated by filling in the *Sal*I site (nucleotide 14,412). To introduce GFP near TM4, a *Bam*HI site was introduced at the desired position (nucleotide 9,851) and GFP sequences including a translational stop site were cloned into the new site, resulting in deletion of 94 amino acids from the carboxyl terminus of UNC-7.

Phenotypic rescue of *unc-7 *by various constructs was assessed by tapping transformed animals on the nose or tail and observing whether or not smooth sinusoidal waves were propagated. *unc-7 *mutants typically cannot move more than one full body-length forward before assuming a severely kinked posture; in the reverse direction they initially produce smooth waves (usually of reduced amplitude compared to wild-type) but quickly display sharp kinks and stop progressing. Forward locomotion was considered rescued if animals could progress forward smoothly for at least several body lengths before showing signs of kinking. Many animals showed fairly long periods of forward movement before suffering a bout of spastic kinking, followed by progressive forward movement. A more precise analysis of the variations involved in rescue of locomotion was beyond the scope of this work.

Determination of the expression pattern of antibody- or GFP-labelled puncta (presumptive UNC-7-containing gap junctions) in various mutant backgrounds was based on examination of at least two (typically more) independently isolated transgenic lines for each condition. Mixed stage animal cultures were antibody-stained, and at least a dozen late larval stage animals (L3, L4) were examined for each line. Pictures used in figures show representative animals expressing constructs on extrachromosomal arrays for each condition.

### *unc-9 *rescue and *innexin::gfp *constructions using long-range PCR

A plasmid encompassing the *unc-9 *gene from exon 4 to the polyadenylation site at nucleotide 12,661 of cosmid R12H7 [21] was constructed in pBluescript (Stratagene, Cedar Creek, TX, USA) extending from the *Kpn*I site at nucleotides 8,959–8,964 to the *Xba*I site at nucleotides 14,519–14,524. A PCR product from nucleotide 4,178 of F09D5 (primer UNC-9A: 5'-TCAGACGCGATGTTTTGTAGGTGGG-3') to nucleotide 9,849 of cosmid R12H7 (Primer UNC-9B: 5'-CCACTTGATGTTTCACGTGTAGCGC-3') was amplified (Expand Long Template PCR System, Roche Diagnostics, Indianapolis, IN, USA) from genomic DNA (14,678 nucleotides total length). The *unc-9 *plasmid and PCR product (50 ng/μl each) were co-injected into *unc-9(fc16) *animals. One rescued line, EH536, was maintained and used for genetic crosses involving *unc-9(fc16)*.

To generate *unc-9::gfp*, an *Nhe*I site was generated at the carboxyl terminus of *unc-9 *(nucleotide 11,703) by amplifying with appropriate primers to include the *Kpn*I site at nucleotides 8,959–8,964 (UNC-9E: 5'-TGCTAGCCACGTCGTGCATTTTTCCTTCTTTATTG-3'; and UNC-9F: 5'-GTTGGGTACCTGCGACGTTCACCGAG-3'). The PCR product was cloned and the isolated *Kpn*I/*Nhe*I fragment then subcloned into a pBS sk- plasmid bearing a GFP (afmx) sequence with an *Nhe*I site at the translational start of GFP. The downstream sequences were PCR-amplified using a primer with an introduced *Spe*I site corresponding to the start of the 3' untranslated region at nucleotide 11,704 (UNC-9C: 5'-TTACTAGTTGACACACCCCAACTTCGTAGC), and a primer at nucleotides 12,836–12,860 (UNC-9D: 5'-CTAGTCTTTGCAGAGCAAGTGAGG-3'). This product was subcloned into the GFP vector as a *Spe*I fragment. To generate a full-length *unc-9::gfp *construct, the 14.7-kb UNC-9A+B PCR product (representing the 5' end of *unc-9*) and the *unc-9::gfp *plasmid were digested separately with *Kpn*I, digests electrophoresed through low-melt agarose (SeaPlaque GTG agarose, FMC, Rockland, ME, USA), and appropriate fragments purified by phenol extraction and ethanol precipitation. Approximately 0.6–1 μg of each fragment was used in a 20-μl ligation reaction, and 1 μl of ligation mix used for long-range PCR amplification with primers UNC-9A+ UNC-9D to produce a final 18.4-kb product. Reaction conditions followed the manufacturer's recommendations, with an annealing temperature of 63°C, an initial 15 minute extension time, and 10+18 cycles. This product was similarly purified in a low-melt agarose gel and used at 1 ng/μl along with 50 ng/μl of the *unc-36(+) *cosmid derivative RIp16 (gift of L Lobel) for microinjection into *unc-36(-) *animals. *unc-9Δ::gfp *was generated by cloning an *unc-9 *PCR-amplified genomic fragment (5'-GGATCCAATGTTGGGTACCTGCGAC-3' and 5'-ACCGGTAGTTCTCCGGCGTGAGTTGC-3') into the *Bam*HI/*Age*I sites of the GFP translational fusion vector pPD95.77 (provided by A Fire). Long-range *unc-9Δ::gfp *PCR products for micro-injections were similarly PCR-amplified from ligations using the UNC-9A primer plus a primer specific to pPD95.77 (5'-TTGCTACAGGCATCGTGGTGTCACG-3').

For *inx-19*, an *Xho*I site was introduced at nucleotides 11,041–11,046 (of cosmid T16H5) in a plasmid that included 1,565 nucleotides 5' and 2,042 nucleotides 3' to this site. A GFP sequence amplified from pPD114.24 (provided by A Fire) with flanking *Sal*I sites and including a translational stop site was subcloned into the *Xho*I site; the resultant construct is predicted to delete 98 amino acids from the carboxyl terminus of INX-19. To generate the full-length GFP construct, the upstream region of *inx-19 *was amplified as a 13.2-kb PCR product (extending to nucleotide 10,910 of T16H5), digested with *Aat*II (nucleotides 9,571–9,576), purified, and ligated to *Aat*II-digested *inx-19::gfp *plasmid for long-range PCR amplification to generate a final product of approximately 16.2 kb. This product was purified and injected at a concentration of 20 ng/μl along with 50 ng/μl RIp16.

For *inx-1*, a 4.3-kb *Bam*HI/*Pst*I fragment encompassing the 3' end of the gene was cloned (nucleotides 13,651-9,385 of cosmid C16E9). GFP (afmx) with a translational stop site was inserted into the *Mlu*I site (nucleotides 11,244-11,239) of this fragment, resulting in the predicted truncation of 15 amino acids from the carboxyl terminus of INX-1. For long-range PCR GFP construction, nucleotides 25,162–12,329 of C16E9 and *inx-1::gfp *plasmid sequences including nucleotides 13,651-9,702 were amplified in separate reactions, digested with *Sex*AI (nucleotides 12,686–12,692), purified, ligated, and amplified to give a final 16.3-kb PCR product. Final product (10 or 30 ng/μl) was co-injected with 50 ng/μl RIp16. For *inx-4*, nucleotides 18,920-17,135 and 17,155-13,093 of cosmid F26D11 were cloned upstream (*Kpn*I/*Nhe*I fragment) and downstream (*Spe*I/*Sac*II fragment) of GFP in a GFP vector (GFP [afmx]-B) by virtue of introduced *Nhe*I, *Spe*I and *Sac*II sites. The resultant construct is predicted to delete 138 amino acids from the carboxyl terminus of INX-4. To generate the long-range PCR GFP product, upstream sequences covering nucleotides 26,894-13,521 of F26D11 were amplified, digested with *Kpn*I (nucleotides 18,901-18,896), the appropriate 8-kb fragment purified and ligated to *Kpn*I-digested *inx-4::gfp *plasmid, and the final 14.6-kb product amplified from the ligation mix. This fragment (20 ng/μl) was co-injected with 50 ng/μl RIp16 for transformation rescue.

### Promoter fusions

A cassette for driving expression of UNC-7S::GFP from heterologous promoters was made by blunt-end cloning a 5.3-kb *Sca*I/*Bam*HI fragment from cosmid F09B12 containing the 3' end of the *unc-7 *gene, inserting GFP amplified from pPD114.24 (provided by A Fire) into the *Sal*I site near the 3' end of the *unc-7 *coding region, and then inserting the 5' coding region with a *Bam*HI/*Sgr*AI fragment obtained from the pCS2+U7S construct used for expression in *Xenopus *ooctyes. The resultant plasmid therefore includes the cDNA sequence for the coding region of UNC-7S plus native 3' untranslated region sequences. The only introns present are those found in the GFP sequences. For all promoter fusions the first intron/exon splice site (or S1 trans-splice site) of the heterologous gene was included on the PCR-amplified promoter fragment.

To show that this construct could rescue forward locomotion in *unc-7 *animals, a 2-kb presumptive promoter region of UNC-7S (*Punc-7S*) was PCR-amplified (primers 5'-AAGGATCCTCACATATTCAAATTCAAGAGATC-3' and 5'-AACCGCGGAACAAATATCAGGAAACTGCGCTC-3') and inserted as a *Sac*II/*Bam*HI fragment into the cassette; the construct (*Punc-7S::unc-7S::GFP*) rescued after micro-injection at a concentration of 25 ng/μl into *unc-7(e5) *animals. A translational fusion of *Punc-7S *in the GFP expression vector pPD95.67 (provided by A Fire) was injected at a concentration of 50 ng/μl and found to express in various head neurons but not in ventral nerve cord motor neurons.

Other presumptive promoters tested included that for *sra-11 *(*Psra-11::unc-7S::gfp*), inserted as a 5.1-kb *Bam*HI fragment (amplified with primers 5'-AGACTTCGACAGTTCAAGCTCTCGG-3' and 5'-GTTGTCATGGATCCTAGCTGAAATATAAGGG-3') and micro-injected at 10–100 ng/μl; *acr-5 *(*Pacr-5::unc-7S;;gfp*), inserted as a 4.4-kb *Sac*II/*Bam*HI fragment (primers 5'-GAGAAAGAGAAGCGCCGCGGCTCAG-3' and 5'-ACTCTGCTACCGGATCCACAGGAGC-3') and micro-injected at 10–25 ng/μl; and *cex-1 *(*Pcex-1::unc-7S::gfp*), inserted as a 1-kb *Sac*II/*Bam*HI fragment (primers 5'-CCGCGGTTTGTCGTGTTCCAAACAGAAGC-3' and 5'-TGCAACAACCAATTCTGAAAGTATAAGATTTGACTG-3') and micro-injected at 100 ng/μl.

Expression of UNC-9 under control of the *acr-5 *promoter (*Pacr-5::unc-9*) was achieved by PCR-amplifying the *unc-9 *coding region (primers 5'-CTAGTCTTTGCAGAGCAAGTGAGG-3' and 5'-CCGTCACGACATGACTTAGGATGAG-3') and cloning this 4.5-kb product into the *Eco*RV site of pBS in an orientation allowing for insertion of *Sac*II/*Bam*HI heterologous promoter fragments. An *unc-9::gfp *expression cassette was made by introduction of GFP (afmx) from the partially rescuing *unc-9::gfp *genomic construct (above).

For many of the heterologous promoter fusions, the plasmid JA-1 [46] was used as a co-injected marker at a concentration of 100 ng/μl. JA-1 is a *col-19::gfp *construct expressed in adults, thus allowing for examination of *unc-7::gfp *localization in larval stages.

### Constructs for *Xenopus *oocyte expression

A full-length version of UNC-7 (pCS2+U7L) was PCR-amplified and cloned into *Bam*HI/*Eco*RI-digested pCS2+ [47,48] with primers 5'-GGATCCATGCTCGGCTCCTCCAGC-3' and 5'-GAATTCTCAGTCTATCGTCCCTTG-3'; a full-length UNC-9 insert was generated by amplifying the coding region from a total RNA preparation using nested primers (final pair 5'-CCGTCACGACATGACTTAGG-3' and 5'-GGTGTGTCACACGTCGTGC-3'), cloning the final PCR product into *Eco*RV-digested, T-tailed pBluescript (Stratagene), and subcloning into pCS2+ as a *Bam*HI/*Xho*I fragment (pCS2+U9). To express UNC-7S, pCS2+U7L was double-digested with *Sna*BI and *Xho*I to remove the *Xba*I site in pCS2+, and a 5'-RACE product representing the 5' end of UNC-7S was inserted as a *Bam*HI/*Xba*I restriction fragment to create pCS2+U7S.

### Gap junction channel formation in *Xenopus *oocytes

cRNA was prepared using SP6 mMessage mMachine kit of Ambion Inc. Oocytes were treated as previously described [49] and coinjected with 20–40 ng of innexin RNA mixed with 4 ng of antisense oligonucleotide directed against nucleotides -5–25 of endogenous *Xenopus *Cx38 [7].

Approximately 48 h after injection, oocytes were stripped of their vitelline membrane and paired overnight in agar wells. Oocytes were continuously bathed in half-strength L15 media (Sigma, St Louis, MO, USA). Junctional currents were assessed using the dual oocyte voltage clamp technique as described previously [49]. Oocytes were clamped at approximately -30 mV using two Geneclamp500 Amplifiers (Axon Instruments, La Jolla, CA, USA). One cell was then pulsed in 10 mV increments to establish *Vj*s up to ± 100 mV. Data were acquired and analyzed using Pclamp8 software (Axon Instruments).

### *In vitro *translation

*In vitro*-translated cRNA (100 ng/μl) was mixed in 50 μl rabbit reticulocyte lysates (Promega, Madison, WI, USA) and incubated (30°C, 90 minutes) with 15 mCi/ml [^35^S]methionine (Amersham, Piscataway, NJ, USA). The *in vitro*-translated products were separated by 12.5% SDS-PAGE and the dried gel imaged with a Phosphoimager (Molecular Dynamics, Sunnyvale, CA, USA).

### *unc-124(hs10) *is an allele of *unc-7*

*unc-124(hs10*) was originally isolated as a cold-sensitive (cs) *unc *mapping to the left arm of chromosome *X *between *dpy-8 *and *unc-10*, at approximately 2.4 cM[50]; *unc-7 *maps to the right arm of *X *at +22 cM. *unc-124 *was reported to display hetero-allelic non-complementation with *unc-7*; that is, *unc-124 *+/+ *unc-7 *animals were reported to be cs Unc [21,50]. We carried out a non-complementation screen for new alleles of *unc-124 *and identified four new mutations. All of these mutations mapped to the right of *unc-3 *(+21.3 cM) and were presumed to be new alleles of *unc-7*. A failure to identify new *unc-124 *alleles led us to sequence the *unc-7 *coding region cloned from *unc-124(hs10*) mutants (strain HH34), and a TGC to TAC change (C238Y) in the predicted second TM domain of UNC-7 was identified. Two other strains carrying the *unc-124(hs10) *mutation have been deposited with the *C. elegans *Genetic Center, and we sequenced the *unc-7 *region in HH123 *unc-2(e55) unc-124(hs10) *and HH132 *dpy-8(e130) unc-124(hs10)*; the same *unc-7 *mutation was identified in both strains.

To verify that *hs10 *mapped to the *unc-7 *locus, HH123 and HH132 hermaphrodites were crossed to N2 males, resultant cross-progeny hermaphrodites back-crossed to N2 males, and wild type and recombinant non-Dpy 'Unc-124' or non-Unc-2 'Unc-124' males grown and scored at 15°C. For *++/unc-2 unc-124 *crosses, 111 wild type and 58 non-Unc-2 Unc-124 males were counted, placing *unc-2 *34.3 cM from *unc-124*, which positions *hs10 *very close to *unc-7 *and quite distant from its original mapped location. For *++/dpy-8 unc-124 *crosses, 132 wild type and 41 non-Dpy Unc-124 males were counted, placing *dpy-8 *23.7 cM from *hs10*, and again positioning *hs10 *very close to *unc-7*.

Was it possible that the original HH34 strain was a double mutant that carried mutations in both *unc-124 *and *unc-7*? To test this, HH34 males were crossed to *dpy-8 lin-2 *hermaphrodites at 25°C. *dpy-8 *maps at position -6.2 cM and *lin-2 *at +7.0 cM. Heterozygous non-Dpy non-Lin hermaphrodite cross progeny were grown at the restrictive temperature (15°C), and non-Dpy Lin segregants were identified and isolated. Broods from these animals grown at 15°C were later examined for the presence of any animals displaying a kinker (Unc-124-like) phenotype. In two separate experiments, a total of 29 non-Dpy Lin animals were isolated and none of them segregated any progeny displaying a kinker phenotype. We conclude that there is no Unc mutation that maps to the left of *lin-2 *in *unc-124(hs10) *strains currently held at the *C. elegans *Genetic Center, and that *hs10 *is an allele of *unc-7*.

### Genetic mosaics identify the AB lineage as the focus of action for *unc-9*

Mosaic analysis of *unc-9 *followed the same strategy used for *unc-7 *[9]. The genetic markers employed were used to distinguish early duplication losses from the founder cell AB (giving rise to nearly all non-pharyngeal neurons), or P_1 _(giving rise to the germ line as well as all but one of the 95 body wall muscle cells). Two different strains were used. SP800 was of genotype *unc-93(e1500) III; unc-9(e101) unc-3(e151) sup-10 (mn219) osm-1(p808) X; mnDp3 (X, f) [unc-9(+) unc-3(+) sup-10(+) osm-1(+)]*. *unc-93(e1500) *confers a gain-of-function rubberband phenotype that is suppressed by the recessive *sup-10 (mn219) *mutation. The focus of action of the *e1500 *rubberband phenotype is the body wall muscles – when the *sup-10(+) *free duplication *mnDp3 *is present in body wall muscles, the animals have a rubberband phenotype due to *unc-93*. If in a rare mitotic event *mnDp3 *is lost in the lineage giving rise to body wall muscles, animals will be wild type due to recessive suppression by *sup-10*. *unc-3 *animals are severe Unc coilers, and *unc-3 *is epistatic to *unc-9*; the focus of action of *unc-3 *is widely distributed in the ABp lineage. The focus of action of *osm-1 *is in sensory neurons associated with amphids and phasmids that also derive from AB. We used SP800 to screen for rare non-Unc-3 non-Unc-93 non-Osm-1 animals. These animals are predicted to maintain *mnDp3 *in the AB lineage (non-Unc-3 and non-Osm-1 phenotypes) but will have lost *mnDp3 *in the P_1 _lineage (non-Unc-93 because loss of *mnDp3 *from body wall muscles allows the recessive *sup-10 *mutation to suppress *unc-93*). Further verification of the loss of *mnDp3 *from P_1 _is the absence of *mnDp3 *from any of the progeny (that is, all progeny are expected to be Unc-3 Osm-1 non-Unc-93). We screened approximately 10,000 animals and identified 7 hermaphrodites that were non-Unc-3 non-Unc-93 non-Osm-1 and gave rise to broods that showed only Unc-3 progeny (*osm-1 *was not scored in the broods). None of these seven animals displayed an Unc-9 phenotype, suggesting that the focus of action of *unc-9 *is not in the P1 derived lineage, and that *mnDp3 *maintained in the AB lineage of these animals rescued the *unc-9 *mutation.

The second strain employed was of genotype *unc-9(e120) daf-6(e1377) sup-10(n983); mnDp3 [unc-9(+) daf-6(+) sup-10(+)]*. The gain-of-function *sup-10(n983) *allele [51] confers a rubberband phenotype with focus of action in body wall muscles [52]; the focus of action of *daf-6 *is in the amphid and phasmid sheath cells that derive from ABp [53], and *daf-6 *animals are defective in the dye-filling (Dyf phenotype) of sensory neurons associated with these structures. This strain gives rise to wild-type (carrying *mnDp3*) and Unc-9 Rubberband (lacking *mnDp3*) progeny. If the *unc-9 *focus of action is derived from AB, we expected to find rare Unc-9 non-Rubberband animals arising from loss of *mnDp3 *only in the AB lineage. Such animals were expected to be non-Rubberband because *mnDp3 *complements the *sup-10 *mutation in the body wall muscles, dye-filling defective because of the ABp focus of *daf-6*, and to give rise to both wild-type and Unc Rubberband progeny because of maintenance of *mnDp3 *in the germline derived from P_1 _descendants. Of approximately 15,000 animals screened, 2 were identified that met the criteria of being Unc-9 non-Rubberband Dyf and giving rise to both wild-type and Unc Rubberband progeny. Additionally, six were identified as Unc-9 non-Rubberband non-Dyf, giving rise to both wild-type and Unc Rubberband progeny. Five of the latter were classified as exhibiting a weaker Unc-9 phenotype. We interpret the latter group as potentially representing losses of mnDp3 within the ABa lineage, or representing a more complex pattern of mosaicism within the ABp lineage that maintains mnDp3 in the amphid and phasmid sheath cells.

Although the focus of action of *unc-9 *cannot be precisely determined from these mosaic experiments, the results are consistent with the interpretation that loss of *unc-9(+) *function from the P_1 _lineage can be tolerated without apparent effects on locomotion, and that the loss of *unc-9(+) *function from within the AB lineage gives rise to the Unc-9 phenotype.

## Abbreviations

cs: cold-sensitive; EM: electron micrograph; GFP: green fluorescent protein; RACE: rapid amplification of cDNA ends.

## Competing interests

The authors declare that they have no competing interests.

## Authors' contributions

TAS carried out all experiments involving *C. elegans *and contributed to writing of the manuscript, IMS and JX carried out electrophysiology experiments in *Xenopus *oocytes and JX contributed to writing of the manuscript, BJN participated in design and coordination of electrophysiological studies and contributed to writing of the manuscript, and JES participated in design and coordination of *C. elegans *experiments and contributed to writing of the manuscript.

## Supplementary Material

Additional file 1 **Anti-UNC-7 staining of *unc-7 *mutants**. Anti-UNC-7 staining of *unc-7 *mutants. Click here for file

Additional file 2 **Expression of other *innexin::gfp *constructs**. Tabulated neuronal innexin expression data. Click here for file

Additional file 3 **Boltzmann fit parameters of (*G*_j∞_/*G*_j0_)/*V*_j _relations**. Tabulated data of Boltzmann fit parameters. Click here for file

Additional file 4 **SDS-PAGE analysis of UNC-7 isoforms in rabbit reticulocytes**. Western blot analysis of UNC-7 isoforms translated in rabbit reticulocytes. Click here for file

Additional file 5 **UNC-7S mosaic analysis**. Genetic mosaic analysis of rescue of forward locomotiion by UNC-7S. Click here for file

## References

[B1] Phelan P, Starich TA (2001). Innexins get into the gap. Bioessays.

[B2] Phelan P (2005). Innexins: members of an evolutionarily conserved family of gap-junction proteins. Biochim Biophys Acta.

[B3] Hua VB, Chang AB, Tchieu JH, Kumar NM, Nielsen PA, Saier MH (2003). Sequence and phylogenetic analyses of 4 TMS junctional proteins of animals: connexins, innexins, claudins and occludins. J Membr Biol.

[B4] Bruzzone R, Hormuzdi SG, Barbe MT, Herb A, Monyer H (2003). Pannexins, a family of gap junction proteins expressed in brain. Proc Natl Acad Sci USA.

[B5] Panchin Y, Kelmanson I, Matz M, Lukyanov K, Usman N, Lukyanov S (2000). A ubiquitous family of putative gap junction molecules. Curr Biol.

[B6] Sasakura Y, Shoguchi E, Takatori N, Wada S, Meinertzhagen IA, Satou Y, Satoh N (2003). A genomewide survey of developmentally relevant genes in *Ciona intestinalis*. X. Genes for cell junctions and extracellular matrix. Dev Genes Evol.

[B7] Barrio LC, Suchyna T, Bargiello T, Xu LX, Roginski RS, Bennett MV, Nicholson BJ (1991). Gap junctions formed by connexins 26 and 32 alone and in combination are differently affected by applied voltage. Proc Natl Acad Sci USA.

[B8] Phelan P, Stebbings LA, Baines RA, Bacon JP, Davies JA, Ford C (1998). *Drosophila *Shaking-B protein forms gap junctions in paired *Xenopus *oocytes. Nature.

[B9] Starich TA, Herman RK, Shaw JE (1993). Molecular and genetic analysis of unc-7, a *Caenorhabditis elegans *gene required for coordinated locomotion. Genetics.

[B10] Rash JE, Dillman RK, Bilhartz BL, Duffy HS, Whalen LR, Yasumura T (1996). Mixed synapses discovered and mapped throughout mammalian spinal cord. Proc Natl Acad Sci USA.

[B11] Condorelli DF, Parenti R, Spinella F, Trovato Salinaro A, Belluardo N, Cardile V, Cicirata F (1998). Cloning of a new gap junction gene (Cx36) highly expressed in mammalian brain neurons. Eur J Neurosci.

[B12] Galarreta M, Hestrin S (2001). Electrical synapses between GABA-releasing interneurons. Nat Rev Neurosci.

[B13] White JG, Southgate E, Thomson JN, Brenner S (1986). The structure of the nervous system of *Caenorhabditis elegans*. Philos Trans R Soc Lond B Biol Sci.

[B14] Bruzzone R, White TW, Paul DL (1994). Expression of chimeric connexins reveals new properties of the formation and gating behavior of gap junction channels. J Cell Sci.

[B15] Chalfie M, White J, Wood WB (1988). The nervous system. The Nematode Caenorhabditis elegans.

[B16] Chalfie M, Sulston JE, White JG, Southgate E, Thomson JN, Brenner S (1985). The neural circuit for touch sensitivity in *Caenorhabditis elegans*. J Neurosci.

[B17] Wicks SR, Rankin CH (1995). Integration of mechanosensory stimuli in *Caenorhabditis elegans*. J Neurosci.

[B18] McIntire SL, Jorgensen E, Kaplan J, Horvitz HR (1993). The GABAergic nervous system of *Caenorhabditis elegans*. Nature.

[B19] Duerr JS, Gaskin J, Rand JB (2001). Identified neurons in *C. elegans *coexpress vesicular transporters for acetylcholine and monoamines. Am J Physiol Cell Physiol.

[B20] Stretton A, Donmoyer J, Davis R, Meade J, Cowden C, Sithigorngul P (1992). Motor behavior and motor nervous system function in the nematode *Ascaris suum*. J Parasitol.

[B21] Barnes TM, Hekimi S (1997). The *Caenorhabditis elegans *avermectin resistance and anesthetic response gene unc-9 encodes a member of a protein family implicated in electrical coupling of excitable cells. J Neurochem.

[B22] WORMBASE. http://wormbase.org.

[B23] Chuang CF, Vanhoven MK, Fetter RD, Verselis VK, Bargmann CI (2007). An innexin-dependent cell network establishes left-right neuronal asymmetry in *C. elegans*. Cell.

[B24] Starich T, Sheehan M, Jadrich J, Shaw J (2001). Innexins in *C. elegans*. Cell Commun Adhes.

[B25] Liu Q, Chen B, Gaier E, Joshi J, Wang ZW (2006). Low conductance gap junctions mediate specific electrical coupling in body-wall muscle cells of *Caenorhabditis elegans*. J Biol Chem.

[B26] Chen B, Liu Q, Ge Q, Xie J, Wang ZW (2007). UNC-1 regulates gap junctions important to locomotion in *C. elegans*. Curr Biol.

[B27] Perkins LA, Hedgecock EM, Thomson JN, Culotti JG (1986). Mutant sensory cilia in the nematode *Caenorhabditis elegans*. Dev Biol.

[B28] Miller DM, Shen MM, Shamu CE, Burglin TR, Ruvkun G, Dubois ML, Ghee M, Wilson L (1992). *C. elegans *unc-4 gene encodes a homeodomain protein that determines the pattern of synaptic input to specific motor neurons. Nature.

[B29] Von Stetina SE, Fox RM, Watkins KL, Starich TA, Shaw JE, Miller DM (2007). UNC-4 represses CEH-12/HB9 to specify synaptic inputs to VA motor neurons in *C. elegans*. Genes Dev.

[B30] Winnier AR, Meir JY, Ross JM, Tavernarakis N, Driscoll M, Ishihara T, Katsura I, Miller DM (1999). UNC-4/UNC-37-dependent repression of motor neuron-specific genes controls synaptic choice in *Caenorhabditis elegans*. Genes Dev.

[B31] Troemel ER, Chou JH, Dwyer ND, Colbert HA, Bargmann CI (1995). Divergent seven transmembrane receptors are candidate chemosensory receptors in *C. elegans*. Cell.

[B32] Landesman Y, White TW, Starich TA, Shaw JE, Goodenough DA, Paul DL (1999). Innexin-3 forms connexin-like intercellular channels. J Cell Sci.

[B33] Stebbings LA, Todman MG, Phelan P, Bacon JP, Davies JA (2000). Two *Drosophila *innexins are expressed in overlapping domains and cooperate to form gap-junction channels. Mol Biol Cell.

[B34] Suchyna TM, Nitsche JM, Chilton M, Harris AL, Veenstra RD, Nicholson BJ (1999). Different ionic selectivities for connexins 26 and 32 produce rectifying gap junction channels. Biophys J.

[B35] Gonzalez D, Gomez-Hernandez JM, Barrio LC (2007). Molecular basis of voltage dependence of connexin channels: an integrative appraisal. Prog Biophys Mol Biol.

[B36] Lehmann C, Lechner H, Loer B, Knieps M, Herrmann S, Famulok M, Bauer R, Hoch M (2006). Heteromerization of innexin gap junction proteins regulates epithelial tissue organization in *Drosophila*. Mol Biol Cell.

[B37] Sulston JE, Horvitz HR (1977). Post-embryonic cell lineages of the nematode, *Caenorhabditis elegans*. Dev Biol.

[B38] Starich TA, Miller A, Nguyen RL, Hall DH, Shaw JE (2003). The *Caenorhabditis elegans *innexin INX-3 is localized to gap junctions and is essential for embryonic development. Dev Biol.

[B39] Angstadt JD, Donmoyer JE, Stretton AO (1989). Retrovesicular ganglion of the nematode *Ascaris*. J Comp Neurol.

[B40] Davis RE, Stretton AO (1996). The motornervous system of *Ascaris *: electrophysiology and anatomy of the neurons and their control by neuromodulators. Parasitology.

[B41] Zheng Y, Brockie PJ, Mellem JE, Madsen DM, Maricq AV (1999). Neuronal control of locomotion in *C. elegans *is modified by a dominant mutation in the GLR-1 ionotropic glutamate receptor. Neuron.

[B42] Tsalik EL, Hobert O (2003). Functional mapping of neurons that control locomotory behavior in *Caenorhabditis elegans*. J Neurobiol.

[B43] Kobayashi Y, Bando T (1950). Studies on the locomotion of *Ascaris suilla *and *lumbricoides *observed in the glass tube, and the influence of Santonin. Proc Japan Acad.

[B44] Sulston J, Hodgkin J, Wood WB (1988). Methods. The Nematode Caenorthabditis elegans.

[B45] Bargmann CI, Avery L (1995). Laser killing of cells in *Caenorhabditis elegans*. Methods Cell Biol.

[B46] Abrahante JE, Miller EA, Rougvie AE (1998). Identification of heterochronic mutants in *Caenorhabditis elegans*. Temporal misexpression of a collagen::green fluorescent protein fusion gene. Genetics.

[B47] Rupp RA, Snider L, Weintraub H (1994). *Xenopus *embryos regulate the nuclear localization of XMyoD. Genes Dev.

[B48] Turner DL, Weintraub H (1994). Expression of achaete-scute homolog 3 in *Xenopus *embryos converts ectodermal cells to a neural fate. Genes Dev.

[B49] Skerrett IM, Merritt M, Zhou L, Zhu H, Cao F, Smith JF, Nicholson BJ (2001). Applying the *Xenopus *oocyte expression system to the analysis of gap junction proteins. Methods Mol Biol.

[B50] Hecht RM, Norman MA, Vu T, Jones W (1996). A novel set of uncoordinated mutants in *Caenorhabditis elegans *uncovered by cold-sensitive mutations. Genome.

[B51] Greenwald I, Horvitz HR (1986). A visible allele of the muscle gene sup-10× of *C. elegans*. Genetics.

[B52] Villeneuve AM, Meyer BJ (1990). The role of sdc-1 in the sex determination and dosage compensation decisions in *Caenorhabditis elegans*. Genetics.

[B53] Herman RK (1987). Mosaic analysis of two genes that affect nervous system structure in *Caenorhabditis elegans*. Genetics.

