# Supplementary material for: Interactions between innexins UNC-7 and UNC-9 mediate electrical synapse specificity in the Caenorhabditis elegans locomotory nervous system
Source: Neural Dev. 2009 May 11;4:16. doi: 10.1186/1749-8104-4-16 (PMC2694797; doi:10.1186/1749-8104-4-16)
Supplement: Additional file 1 — Anti-UNC-7 staining of unc-7 mutants. Anti-UNC-7 staining of unc-7 mutants. [file 1749-8104-4-16-S1.doc]

**Additional File 1: anti-UNC-7 antibody staining of *unc-7* mutants**.

Expression of UNC-7 in *unc-7* mutants could be categorized into 4 classes (Table 1). Severe mutants *e5* and *e42* lack the antigenic site and showed no anti-UNC-7 reactivity. Hypomorphic mutants *e65* and *mn384* showed a WT staining pattern, as did cold-sensitive (cs) *hs10* mutants at both 15^o^ and 25^o^C. A third class, including *mn409* and cold-sensitive *hs9* (15^o^ and 25^o^C), showed severely reduced staining that localized to cell bodies, especially at lower temperatures. Both of these mutations affect conserved prolines, and accumulation of protein is consistent with a hypothesis that cs proline mutations in the related innexin *unc-9* may affect protein translocation [1]. The final class (*mn382* and *mn383*) showed strong expression in the nerve ring, retrovesicular ganglion, and tail, but ventral and dorsal nerve cord expression was severely reduced. *mn382* is a translocation that separates the *unc-7* coding region from its upstream promoter, and *mn383* removes approximately 6 kb within intron 1 (Figure 2A). Weaker mutants *e133* and *e139* also represent this class.

1. Barnes TM, Hekimi S**: The Caenorhabditis elegans avermectin resistance and anesthetic response gene unc-9 encodes a member of a protein family implicated in electrical coupling of excitable cell**s*. J Neuroche*m 1997**, 6**9:2251-2260.
