# Supplementary material for: Interactions between innexins UNC-7 and UNC-9 mediate electrical synapse specificity in the Caenorhabditis elegans locomotory nervous system
Source: Neural Dev. 2009 May 11;4:16. doi: 10.1186/1749-8104-4-16 (PMC2694797; doi:10.1186/1749-8104-4-16)
Supplement: Additional file 2 — Expression of other innexin::gfp constructs. Tabulated neuronal innexin expression data. [file 1749-8104-4-16-S2.doc]

**Additional file 2:**

**Expression of other neuronal *innexin::gfp*** constructs

| INX::GFP | Neuron | GJ partners |  |
| --- | --- | --- | --- |
| INX-1 | RIG | AVK, AQR, BAG, OLL,  OLQ, RIB, URY, RIG |  |
|  | DA, DB | AVA, AVB, DA, DB |  |
|  | + body wall muscle; pharyngeal muscle | |  |
| INX-4 | ADF* | RIH, **ADA**, AIA, ADF |  |
|  | AFD* | AIB, AFD |  |
|  | AQR* | AVK, PVP, **RIG** |  |
|  | ASI* | AIA, ASI |  |
|  | AWA* | AIA, AIZ, AWA |  |
|  | AWB* | **AUA**, RMG, AWB |  |
|  | AWC* | none described |  |
|  | BAG* | **RIG**, RIR, BAG |  |
|  | FLP* | AVD, RIH, FLP |  |
|  | OLL* | IL1, **RIG**, OLL |  |
|  | ADA | **ADF**, ASH, AVD, PVQ, ADA |  |
|  | AIN | ASG,**AUA**,AIN |  |
|  | AUA | **AIN**,**AWB**,URX,AUA |  |
|  | RIG | AVK,**AQR**,**BAG**,**OLL**  OLQ,RIB,URY,RIG |  |
|  | RIH | **ADF**,CEP,**FLP**,OLQ |  |
|  | RIP | I1,RMED |  |
|  | DVC | AVL,PVP,VD1 |  |
|  | + m1 pharyngeal muscles  +5 tail neurons  +6 addl. pr. head neurons? | |  |
|  |  |  |  |
|  |  |  |  |
| INX-19 | AVB | DBn,VBn, DVA, PVN, RIB, RID, SDQ, SIBV, AVB |  |
|  |  |  |  |
|  | OLL | IL1,RIG,OLL |  |
|  | AVA  (w) | ASn, DAn,VAn, SAB, PVC, RIM, URY, AVA, (DB5) |  |
|  | RIP(w) | I1, RMED |  |
|  | AIN(w) | ASG, AUA, AIN |  |
|  | BDU(w) | none |  |

*Ciliated neurons; **Bold**=Gap junction partners expressing same INX;

(w)=weak expression ; (cell)= occasional gap junction?
