# Supplementary material for: Interactions between innexins UNC-7 and UNC-9 mediate electrical synapse specificity in the Caenorhabditis elegans locomotory nervous system
Source: Neural Dev. 2009 May 11;4:16. doi: 10.1186/1749-8104-4-16 (PMC2694797; doi:10.1186/1749-8104-4-16)
Supplement: Additional file 3 — Boltzmann fit parameters of (Gj∞/Gj0)/Vj relations. Tabulated data of Boltzmann fit parameters. [file 1749-8104-4-16-S3.doc]

**Additiional file 3:**

**Boltzmann fit parameters of (*G*_j_/ *G*_j0_)/*V*_j_ relatio**ns

|  | | *A* | *V*_0_ | *G*_jmin_ |
| --- | --- | --- | --- | --- |
| UNC-7S | Positive *V*_j_ | 0.077±0.009 | 52.7±3.2 | 0.29±0.06 |
|  | Negative *V*_j_ | 0.075±0.011 | -53.8±3.6 | 0.24±0.05 |
| UNC-7L | Positive *V*_j_ | 0.077±0.005 | 47.4±1.9 | 0.26±0.04 |
|  | Negative *V*_j_ | 0.073±0.002 | -50.0±2.4 | 0.25±0.02 |
| UNC-7S/UNC-9 | Positive *V*_j_ | 0.114±0.008 | 38.5±3.3 | 0.09±0.02 |
| UNC-7L/UNC-9 | Positive *V*_j_ | 0.097±0.012 | 37.4±1.6 | 0.13±0.02 |
| UNC-7S/UNC-7L | Positive *V*_j_ | 0.077 ±0.006 | 46.3±3.5 | 0.14±0.02 |
|  | Negative *V*_j_ | 0.076±0.018 | -44.9±3.8 | 0.36±0.10 |
| UNC-7S/UNC-7L+9 | Positive *V*_j_ | 0.079±0.005 | 43.5±1.0 | 0.15±0.02 |
|  | Negative *V*_j_ | 0.066±0.003 | -50.4±0.6 | 0.35±0.03 |

(*G*_j∞_/ *G*_j0_)/*V*_j_ relations for one polarity of voltage are fit by a Boltzmann relation of the form: *G*_j∞_=(*G*_jmax_- *G*_jmin_)/{1+exp[*A*(*V*_j_- *V*_0_)]}+ *G*_jmin_. *V*_0_ is the voltage at which *G*_j∞_ is half way between *G*_jmin_ and *G*_jmax_. *A = zq/kT* is a cooperativity constant reflecting equivalent charge movement during gating, where *z* is the number of charges moving through the entire applied voltage, *q* is the charge on the electron, and *kT* has its usual meaning. No Boltzman parameters could be determined for UNC-9 homotypic pairings, or for negative polarities of any UNC-7 pairings with UNC-9, due to insufficient gating response, or because the gating response was only partially completed over the voltage range recorded.
