# Supplementary material for: Interactions between innexins UNC-7 and UNC-9 mediate electrical synapse specificity in the Caenorhabditis elegans locomotory nervous system
Source: Neural Dev. 2009 May 11;4:16. doi: 10.1186/1749-8104-4-16 (PMC2694797; doi:10.1186/1749-8104-4-16)
Supplement: Additional file 4 — SDS-PAGE analysis of UNC-7 isoforms in rabbit reticulocytes. Western blot analysis of UNC-7 isoforms translated in rabbit reticulocytes. [file 1749-8104-4-16-S4.doc]

**Additonal file 4: SDS-PAGE analysis of UNC-7 isoforms in rabbit reticulocytes**

Autoradiogram of an SDS-polyacrylamide gel of in vitro translation products from rabbit reticulocyte lysates for: (1) UNC-7S; (2) UNC-7L; (3) UNC-7L(M121L); (4) UNC-9. MW markers are indicated on the left. The arrow indicates a product (UNC-7SR) likely derived from the internal ATG in UNC-7S and L that has been mutated in UNC-7L(M121L). A small band above this may represent a degradation product of UNC-7L, as it is seen in both wild type and mutant UNC-7L forms.

We tested each of the UNC mRNAs used in our oocyte studies in the rabbit reticulocyte expression system to ensure that the RNA was functional. Each produced an expected major product, albeit smaller than predicted (~40 kD for UNC-7S, 46 kD for both UNC-7L and UNC-7L[M121L], and 30 kD for UNC-9; predicted sizes for UNC-7S, UNC-7L, UNC-7SR and UNC-9 are 53, 60, 47, and 45 kD, respectively), and some smaller bands. Notably, UNC-7S and L both produced a 35-kD band that is likely to represent UNC-7SR, as this band was not produced from UNC-7L(M121L) where the internal translation start site predicted to produce UNC-7SR was eliminated. A product slightly larger than 35 kD was detected in the UNC-7L(M121L) extract, but this could represent a degradation product, as observed with UNC-7L. These results suggest that currents recorded for both UNC-7 isoforms may include a large UNC-7SR component, consistent with its isolation in worm protein extracts (Figure 3H).
