# Supplementary material for: Interactions between innexins UNC-7 and UNC-9 mediate electrical synapse specificity in the Caenorhabditis elegans locomotory nervous system
Source: Neural Dev. 2009 May 11;4:16. doi: 10.1186/1749-8104-4-16 (PMC2694797; doi:10.1186/1749-8104-4-16)
Supplement: Additional file 5 — UNC-7S mosaic analysis. Genetic mosaic analysis of rescue of forward locomotiion by UNC-7S. [file 1749-8104-4-16-S5.doc]

**Additional file 5: UNC-7S mosaic analysis**


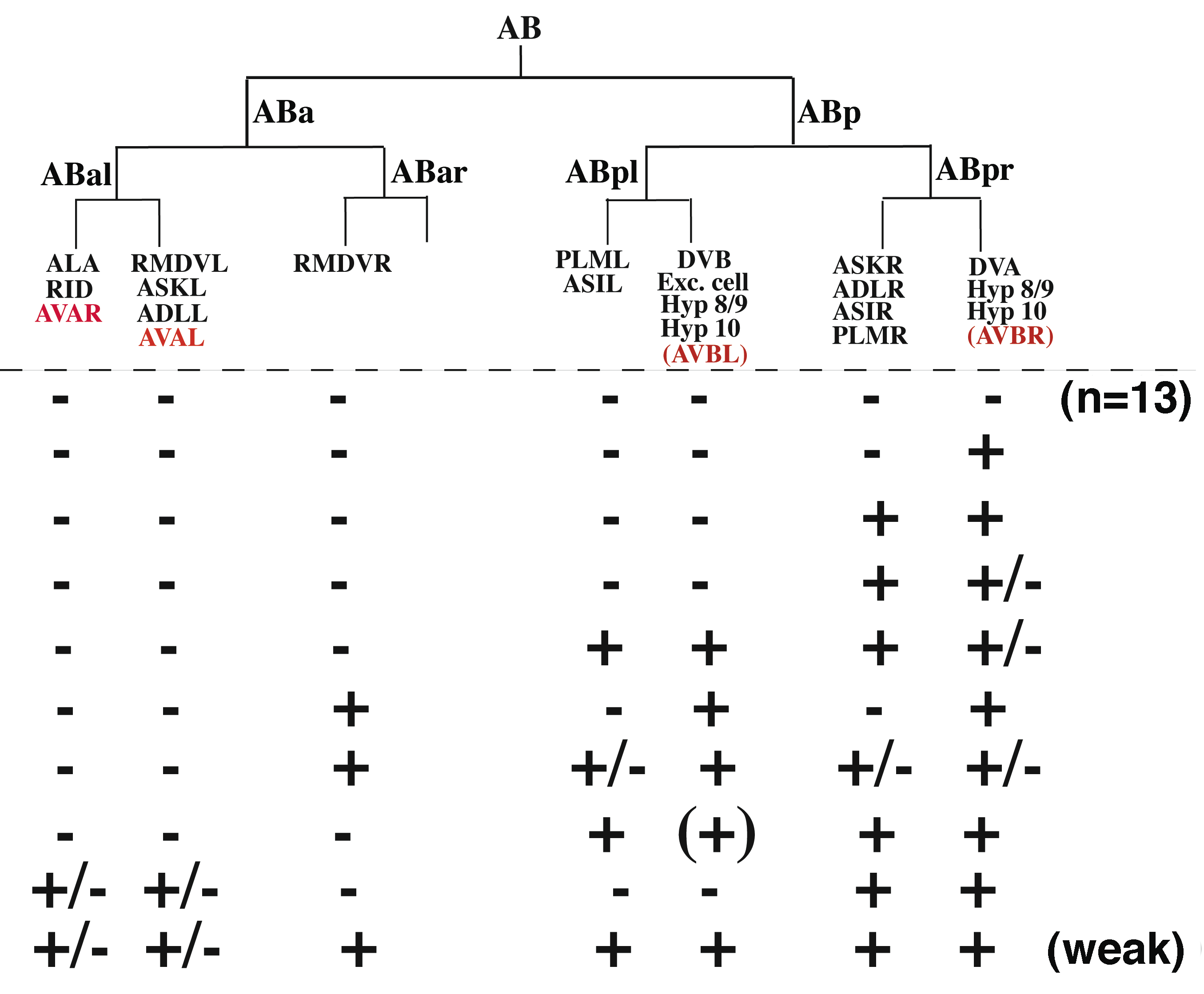


“**+**” indicates all cells scored within that lineal branch were GFP(+); “**+/-**“ indicates cells varied within that lineal branch; “(+)” indicates that cells usually scored were GFP(+), but other cells in that lineage were noted to be GFP(-). AVAL and AVAR were GFP(-) in all cases.

*unc-7(e5)* animals were rescued for forward locomotion with an extrachromosomal array containing *unc-7S* and *sur-5::gfp* [1]. GFP(+) animals that were forward Unc were identified. The GFP status of 21 cells representing different lineages was scored; to minimize effects of photobleaching, cells were chosen that could be quickly and reliably identified, including both AVAs. (AVBs were difficult to score without extensive scrutiny and usually not attempted.) In all cases both AVAL and AVAR (ABa lineage) had lost the array. In 6 control (non-Unc) animals, only a total of 3 cells in our reference group had lost the array. We concluded that loss in both AVAs is necessary to produce a forward Unc phenotype, i.e. UNC-7S rescues forward locomotion in *unc-7(e5)* animals at least partly through expression in AVA. Because the GFP status of AVBs can only be inferred by reference to other cells related in lineage, it is possible that additional loss of the array in at least one AVB must also accompany AVA loss to produce a forward Unc animal. Indeed, one animal that showed AVA loss but maintained the array in the ABp lineage (both AVBs) exhibited only a weak forward Unc phenotype. To verify an AVA requirement, we ablated the AVA interneuron pairs in *unc-7(e5)* L1 stage animals rescued for forward locomotion by *unc-7S*--8/8 animals showed loss of sustained coordinated forward locomotion after ablation, in support of the genetic mosaic results. We conclude that UNC-7S over- or mis-expression in AVA alters its function such that it is necessary for rescue of forward locomotion in *unc-7(e5)*.

This was a surprising result since the original genetic mosaic analysis indicated an ABp focus of action for *unc-7(+)*. This original analysis utilized free duplications carrying the entire *unc-7* locus, and phenotypic Unc-7 animals were isolated that had lost the duplication in ABp lineage but maintained it in ABa; therefore animals expressing *unc-7(+)* in AVA but neither in AVB nor motor neurons were phenotypically Unc. Mosaic analysis in the present study involved restricted interneuron expression of UNC-7S on multi-copy extrachromosomal arrays in a probably-miswired *unc-7(e5)* background. In this background the expression levels of UNC-7S provided by a multi-copy array may change the wiring pattern of AVA, either through an early developmental change or directly as a gap junction component in the mature nervous system. How might AVA take control of forward locomotion in *e5* mutants? Reconstruction of *e5* showed that AVA made gap junctions with B motor neurons; expressing sufficient UNC-7S in AVA may provide a subunit that makes these channels functional, or may allow a greater number of channels to be formed. Alternatively AVA might act through gap junctions it shares with PVC interneurons, which synapse directly onto B motor neurons. A third possibility is that AVA adopts a more AVB-like fate developmentally as a result of strong UNC-7S expression.

1. Yochem J, Gu T, Han M**: A new marker for mosaic analysis in Caenorhabditis elegans indicates a fusion between hyp6 and hyp7, two major components of the hypodermi**s*. Genetic*s 1998**, 14**9:1323-1334.
